# Plant-Derived Peptides with Neuroprotective Activity: Advances and Perspectives in the Prevention of Neurodegenerative Diseases

**DOI:** 10.1021/acsomega.6c00495

**Published:** 2026-04-08

**Authors:** Maria Eduarda Maia, Matheus Carvalho, Cleyton Sousa Gomes, Milena Arruda, Ana Júlia Antunes de Magalhães, Davi Farias

**Affiliations:** † Laboratory for Risk Assessment of Novel Technologies, Department of Molecular Biology, 28097 Federal University of Paraiba, João Pessoa 58050-085, Brazil; ‡ Post-Graduation Program in Natural and Synthetic Bioactive Products, Federal University of Paraíba, 58051-900 João Pessoa, Brazil; § Post-Graduation Program in Biochemistry, Department of Biochemistry and Molecular Biology, Building 907, Campus Pici, 28121 Federal University of Ceará, 60455-970 Fortaleza, Brazil

## Abstract

Neurodegenerative diseases, such as Alzheimer’s, Parkinson’s, Huntington’s, and amyotrophic lateral sclerosis, represent an increasing global public health challenge, driven by population aging and the lack of effective curative therapies. In this context, plant-derived peptides have emerged as promising bioactive compounds due to their multitarget neuroprotective properties and favorable safety profiles. This review provides a comprehensive overview of plant peptides with reported activity against neurodegeneration, highlighting their natural sources, biological activities, and mechanisms of action. Evidence from *in vitro* and *in vivo* models indicates that these peptides act through multiple complementary pathways, including attenuation of oxidative stress, modulation of neuroinflammation, regulation of apoptosis, preservation of mitochondrial function, and inhibition of toxic protein aggregation. Additionally, several peptides have been shown to enhance synaptic plasticity, modulate neurotransmission, and regulate ion channel activity, suggesting beneficial effects on neuronal communication and cognitive function. Some studies explored structural modifications, such as the introduction of specific residues or glycosylation, which have resulted in greater stability and enhanced efficacy against oxidative insults. Overall, plant-derived peptides demonstrate consistent neuroprotective effects and low toxicity; however, challenges related to the blood–brain barrier, bioavailability, and the understanding of molecular mechanisms must still be overcome to enable their clinical application.

## Graphical abstract

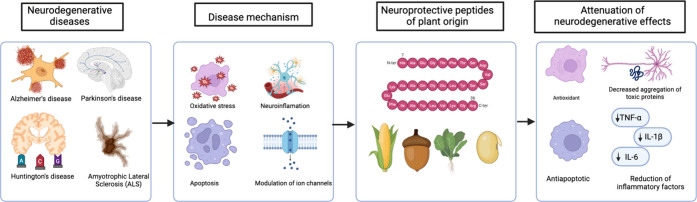

## 1 Introduction

With the increase in life expectancy, neurodegenerative diseases have become one of the greatest challenges of modern medicine due to their high prevalence, debilitating functional progression, and lack of effective curative therapies.^1^ Among these, Parkinson’s disease (PD), Alzheimer’s disease (AD), and Huntington’s disease (HD) stand out for their central feature: the progressive degeneration of neurons in specific brain regions, which significantly compromises patients’ quality of life.^2,3^

These pathologies share common pathophysiological mechanisms, including exacerbated oxidative stress, chronic inflammation, mitochondrial dysfunction, accumulation of toxic protein aggregates such as beta-amyloid (Aβ) and alpha-synuclein, and dysregulation of intracellular calcium.^4,5^ These processes promote progressive neuronal damage and are directly associated with clinical manifestations such as cognitive decline, emotional and behavioral disturbances, memory loss, and motor dysfunction.^6^

Currently, the management of neurodegenerative diseases combines pharmacological and nonpharmacological approaches. In AD, cholinesterase inhibitors such as donepezil are used to improve cognitive function, although they fail to prevent disease progression. In PD, levodopa is widely used to restore dopamine levels and alleviate motor symptoms,^7,8^ nonpharmacological strategies, including physical therapy, cognitive stimulation, and lifestyle modifications, are also applied as complementary interventions but, alone, are insufficient to halt neurodegeneration.^9^

Although current treatments can relieve symptoms and slow disease progression, there are still no therapies capable of stopping or reversing the course of neurodegeneration, highlighting the urgent need for innovative and more effective approaches. In this context, natural-origin therapies have been increasingly investigated as promising alternatives for the treatment of neurodegenerative diseases due to their wide availability, lower toxicity, and multifactorial mechanisms of action.^10–12^ Recent studies have emphasized the potential of plant-derived molecules, including peptides, polyphenols, and other bioactive compounds, to influence oxidative stress, mitochondrial function, protein aggregation, and other processes contributing to neuronal damage.^12,13^

Among these, plant-derived bioactive peptides have attracted considerable attention, as they commonly exhibit antioxidant, anti-inflammatory, and neuroprotective properties capable of modulating key pathways involved in neurodegeneration.^14^ The main mechanisms modulated by plant peptides include the reduction of oxidative stress through free radical neutralization, attenuation of chronic inflammatory responses via regulation of pro-inflammatory cytokines,^15^ the protection of mitochondrial function, and inhibition of the formation and aggregation of toxic proteins such as beta-amyloid and alpha-synuclein.^16,17^

Therefore, this study aimed to critically analyze the available literature on the neuroprotective effects of plant-derived bioactive peptides against the pathophysiological mechanisms associated with neurodegenerative diseases. To achieve this, the main neuroprotective mechanisms modulated by these peptides were examined, including oxidative stress, inflammation, mitochondrial dysfunction, and protein aggregation. Furthermore, the plant sources of these peptides, the extraction and purification techniques employed, the *in silico*, *in vitro*, and *in vivo* assays used to evaluate their efficacy, and their safety profiles were analyzed. These insights provide a solid foundation for understanding the potential of plant peptides in neuroprotection and guide future research in this field.

## 2 Materials and Methods

The PubMed, Web of Science (WoS), Scopus, and ScienceDirect databases were used to search for scientific articles through a comprehensive strategy using the following keyword combinations: (i) “Bioactive peptides” AND “neuroprotection”; (ii) “Bioactive peptides” AND “neurodegenerative”; (iii) “Plant peptides” AND “neuroprotection”; and (iv) “Walnut peptides” AND “neuroprotective”. The searches were conducted between April and July 2025. No restrictions were applied regarding the year of publication; all studies available in the consulted databases were considered.

The following inclusion and exclusion criteria were applied to select the most relevant studies:

Inclusion criteria: studies investigating plant-derived or bioinspired peptides with potential neuroprotective effects, published in English.
Exclusion criteria: review articles, gray literature (*e.g.*, dissertations, theses, and conference abstracts), and studies focusing on animal-, microbial-, or synthetic-derived peptides.

Included articles were analyzed for relevant data, including the plant source and peptide sequences, extraction and purification methods, *in silico*, *in vitro*, and *in vivo* experimental models used, observed neuroprotective mechanisms (*e.g.*, antioxidant or anti-inflammatory activity), and information regarding safety or toxicity profiles.

## 3 Results and Discussion

### 3.1 Overview

The searches in PubMed, WoS, Scopus, and ScienceDirect yielded 90, 132, 109, and 1,989 records, respectively. After the inclusion and exclusion criteria were applied and duplicates were removed, a total of 41 unique articles were selected for analysis (Figure 1).

**Figure 1:**
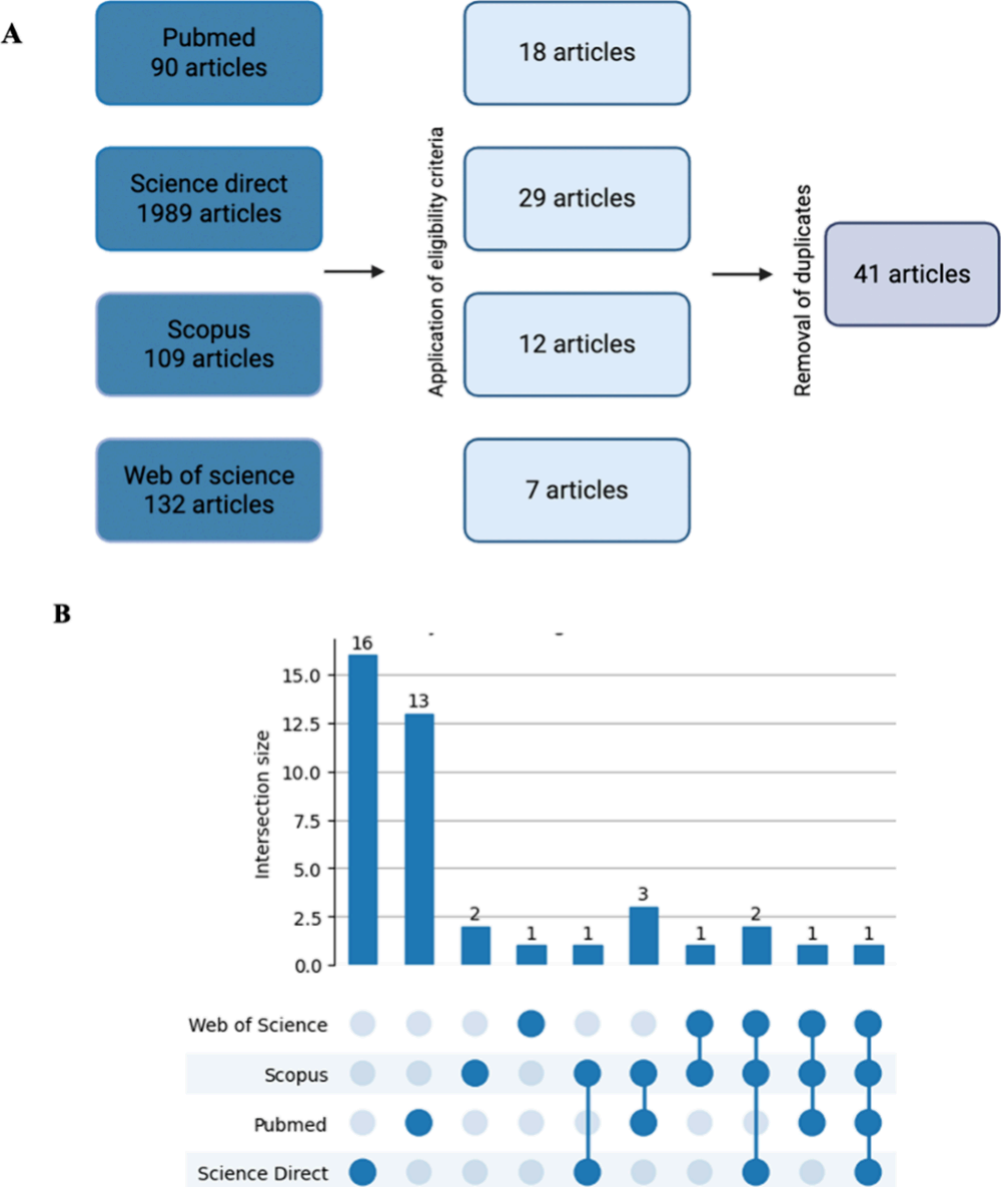
Methodological approach for a systematic review on the use of plant peptides against the pathophysiological mechanisms of neurodegenerative diseases. (A) Article selection process from the databases after applying eligibility criteria and removing duplicates. (B) UpSet plot showing article overlap across the selected databases.

Table 1 summarizes the main characteristics of the selected studies investigating plant-derived peptides with potential neuroprotective activity. For each article, the DOI, authors, peptide sequence, presence of synthetic analogs, and plant sources are provided. The techniques used for extraction and purification are also described, along with the *in silico*, *in vitro*, and *in vivo* assays performed and their respective outcomes. Additionally, the investigated neuroprotective mechanisms and peptide safety profiles are highlighted.

**Table 1:** Studies on Plant-Derived Peptides with Neuroprotective Potential, Their Sources, Sequences and Mechanisms of Action

ref	peptide sequence	plant source	sample preparation	in silico tests	results
^18^	TWLPLPR (TW-7)	walnut	protein precipitation with acetonitrile, followed by centrifugation and HPLC analysis with spectrophotometric detection		
^42^	SGGY	walnut	protein was extracted with NaOH, purified by ultrafiltration, gel filtration chromatography, and RP-HPLC; peptides identified via UPLC-QTOF-MS with electrospray ionization		
^76^	WSREEQEREE and ADIYTEEAGR	walnut	peptides were extracted and purified via simulated gastrointestinal digestion, followed by UPLC-QTOF-MS/MS analysis. Peptides were identified based on fragment detection in extracted ion chromatograms	docking molecular evaluated peptide–Keap1 binding using AutoDock Tools and Vina; structures came from RCSB and ChemBio3D	peptides strongly interacted with Keap1 via multiple hydrogen bonds and pi interactions, indicating effective Keap1 inhibition
^77^	not identified (peptide mixture/hydrolysate	walnut	the walnuts were ground, and proteins were extracted using Tris buffer, then precipitated at isoelectric pH and lyophilized		
^41^	WEKPPVSH	walnut	the peptide was obtained by enzymatic hydrolysis, purified by chromatography and ultrafiltration, and identified by HPLC-MS/MS		
^20^	TWLPLPR (TW-7)	walnut		the TW-7 structure was optimized using GROMACS and docked to C1q via AutoDock. The best complex was analyzed with PyMOL and LigPlus	the molecular docking analysis showed that TW-7 has high binding affinity to the C1q phosphatidylserine (PS) recognition region, suggesting it may competitively inhibit C1q binding to synapses
^21^	TWLPLPR (TW-7)	walnut		molecular docking was performed to predict interactions between TW-7 and MMP-9 using PEP-FOLD3, AlphaFold2, and Schrödinger Maestro	the peptide TW-7 stably bound to the active site of MMP-9, forming a complex through hydrogen and hydrophobic interactions. This binding inhibits MMP-9’s proteolytic activity, protecting the integrity of the blood-brain barrier
^48^	not identified (peptide mixture/hydrolysate	walnut			
^22^	GGW, VYY, and LLPF	walnut	walnut protein hydrolysate (WPHL) was fractionated into low-molecular-weight fractions by chromatography, and neuroprotective peptides were identified by UPLC-QTOF-MS/MS		
^23^	LPF, GVYY, and APTLW	walnut	walnut protein as hydrolyzed, and the low-weight fraction isolated by ultrafiltration. WPHL was purified into six fractions by chromatography. The peptides were identified by UPLC-Q-TOF-MS and analyzed with Mascot and de novo methods		
^19^	TWLPLPR YVLLPSPK, and KVPPLLY,	walnut			
^24^	YVLLPSPK.	walnut			
^62^	PPKNW (PW-5)	walnut	degreased walnut dregs were hydrolyzed with alkali protease at 55 °C for 4 h, then heated to stop the reaction. After centrifugation, the supernatant was freeze-dried to obtain walnut protein hydrolysate (WPH)		
^66^	SGGY	walnut			
^74^	Not identified (peptide mixture/hydrolysate)	walnut	the peptide was mixed with deionized water, and then the enzymes were inactivated. The mixture was centrifuged, the supernatant was collected, and lyophilized	computational analysis was used to predict the binding of peptides to AChE and Keap1, key targets in neurotransmission and antioxidant defense	identification of 20 promising peptides showing the best binding affinities (−9.8 and −8.0 kcal/mol), indicated as potential inhibitors of AChE and Keap1
 ^10^	not identified (peptide mixture/hydrolysate)	walnut		in silico hydrolysis of walnut proteins with various enzymes revealed high release of arginine-rich peptides. Their neuroprotective potential was predicted based on positive charge, cell permeability, and interaction with neurochemical targets using PeptideRanker	twenty promising Arg-containing peptides (2–6 residues) were identified, with repeated sequences in walnut proteins and high activity scores. Most showed strong cell-penetrating ability
^78^	not identified (peptide mixture/hydrolysate)	walnut	the peptide was extracted from defatted walnut flour. After centrifugation, the liquid was concentrated, dialyzed, and lyophilized		
^67^	not identified (peptide mixture/hydrolysate)	walnut			
^26^	YSPYPQ and PLYSN	soybean	hydrolysate was extracted with enzymes, purified by ultrafiltration and chromatography, and peptides identified by LC-MS/MS and PeaksStudio analysis	peptide sequences were docked to monoamine oxidases using HPEPDOCK to identify potential MAO inhibitors	peptides YSPYPQ and PLYSN showed strong potential to inhibit MAO-A and MAO-B, with interactions analyzed via molecular docking
^25^	SWGEDWGEIW	soybean	the peptide was extracted with acetonitrile, methanol, and water. It was purified by gel filtration chromatography and identified by mass spectrometry		
^72^	VHVV	soybean			
^75^	not identified (peptide mixture/hydrolysate)	soybean			
^28^	not identified (peptide mixture/hydrolysate)	black soybean			
^29^	not identified (peptide mixture/hydrolysate)	black soybean			
^79^	not identified (peptide mixture/hydrolysate)	black soybean	black soybean powder was defatted with *n*-hexane, then extracted in water at pH 8.0. After centrifugation, the supernatant was acidified to pH 4.5 to precipitate proteins, which were collected, frozen, and lyophilized. The protein content of the BSP was 90.34% ± 1.04 (Kjeldahl method)	the 7S and 11S protein sequences from black soybean were analyzed using BIOPEP-UWM to predict antioxidant peptides. The release frequency and relative occurrence of these fragments were calculated based on enzymatic simulation	the simulation indicated that subtilisin, chymotrypsin, papain, and bromelain are effective in releasing antioxidant peptides from 7S globulin and 11S glycinin, with subtilisin showing the highest antioxidant potential
^30^	not identified (peptide mixture/hydrolysate)	sesame	extracted with acetate buffer, purified by ion-exchange chromatography, lyophilized, and peptides were identified by HPLC		
^31^	not identified (peptide mixture/hydrolysate)	sesame cake	extracted with acetate buffer and purified by ion exchange chromatography		
^16^	not identified (peptide mixture/hydrolysate)	sesame	sesame cake peptides were extracted with acetate buffer, purified by ion-exchange chromatography, lyophilized, and identified by Q-Exactive MS after HPLC-C18		
^38^	not identified (peptide mixture/hydrolysate)	*Achyranthes bidentata* Blume	extracted using ethanol, purified by HPLC, and analyzed by LC–MS/MS. Its structure was studied by NMR and refined computationally		
^37^	not identified (peptide mixture/hydrolysate)	*Achyranthes bidentata* Blume	the peptide was extracted from roots by ammonium sulfate fractionation, dialysis, and chromatography using C18 and YMC columns. Purity was confirmed by Q-TOF-MS		
^55^	YYLLVR	hazelnut		targets predicted for hypertension and Alzheimer’s; PPI, GO/KEGG analysis; molecular docking and visualization performed	in silico analysis identified 29 targets, mainly RAS proteins (ACE, ACE2, AGTR1); strong binding suggests neuroprotection in hypertension-induced neuronal injury
^17^	NKFGKFF (NF), GGPFKSPF (GF), and RPVLGGSSTFPYP (RP)	pea	hydrolysate made with Flavourzyme at 50 °C for 6h, purified by Superdex Å, peptides identified by LC/MS/MS with electrospray ionization	--	--
^39^	APCPNR, LGLFR, LIPQE, and SISWSS	*Clitoria ternatea*	peptides extracted from flowers using pH buffers, purified by ultrafiltration and identified by HPLC and MALDI-TOF MS	molecular docking analysis with amyloid beta and acetylcholinesterase	the peptides strongly bound amyloid beta, suggesting antiaggregation activity, and also bound ache, indicating inhibitory potential
^34^	not identified (peptide mixture/hydrolysate)		peptide extraction from chia seeds was performed by enzymatic hydrolysis using pepsin and pancreatin, followed by ultrafiltration to separate peptide fractions		
^61^	ALLTLSPLGPA and SPLGPA	corn	hydrolyzed with pepsin; the hydrolysate was centrifuged and lyophilized. Peptides were identified by LC-MS/MS and analyzed using PEAKS Studio	molecular docking with AutoDock Vina evaluated peptide interactions with the POP enzyme using the substrate Z-gly-Pro-pNA to determine binding affinity	peptides inhibit POP by forming hydrogen bonds at sites outside the active site, causing conformational changes that lead to enzyme inhibition
^56^	GPETAFLR	*Lupinus angustifolius* L.	the peptide was produced by enzymatic hydrolysis of lupin protein isolate, purified by ultrafiltration and chromatography, identified by nano-HPLC–mass spectrometry and synthesized		
^33^	RFV	wheat germ			
^32^	EM-1(YPWF), EM-2(YPFF), R-5(YPLDL), and R-6(YPLDLF)	spinach			
^80^	R-6(YPLDLF)	spinach			
^36^	not identified (peptide mixture/hydrolysate)	fermented rice	peptides were extracted using centrifugation, filtration, dialysis, and ultrafiltration, then purified by chromatography and HPLC, and identified by MALDI-TOF/TOF mass spectrometry		
^35^	DFVADHPFLF (DF-10), HGQNFPIL (HL-8), and RDFPITWPW (RW-9)	oat	defatted oat bran flour was mixed with water, adjusted to alkaline pH, stirred, and centrifuged to remove precipitate. The supernatant was then acidified, centrifuged again, and proteins were precipitated	screening through peptidomic analysis and molecular docking simulation to evaluate peptide binding affinity with neuroprotection-related targets, including Keap1 and cholinergic receptors	identification of 25 promising peptides; among them, DF-10, HL-8, and RW-9 were selected based on their high inhibitory activity against Keap1 and predicted ability to modulate antioxidant and anti-inflammatory pathways

ref	in vitro tests	results	in vivo tests	results	neuroprotection mechanisms
^18^	primary hippocampal neurons from mice were cultured in MEA plates, treated with Aβ 25–35 and TW-7, and analyzed for neuronal activity. Immunofluorescence was used to assess the expression of MAP2, SYP, PSD95, and vGlut1.	reduced Aβ-induced neuronal hyperexcitability, preserved dendrites, and normalized synaptic proteins, indicating neuroprotection in Alzheimer’s disease			reduces Aβ-induced neuronal hyperactivity by modulating glutamate release, inhibiting vGlut1 overexpression, and restoring synaptic plasticity
^42^	antioxidant activity of peptides was assessed in SH-SY5Y cells by ABTS radical scavenging, ORAC value, and protection against H_2_O_2_-induced cytotoxicity	the peptide showed significant ABTS radical scavenging activity and high ORAC value. Additionally, peptide SGGY effectively protected SH-SY5Y cells from H2O2-induced oxidative damage			reduction of oxidative stress in SH-SY5Y cells
^76^	assays evaluated PC12 cell viability, ROS-induced damage, LDH release, apoptosis-related proteins (Bax, Bcl-2), antioxidant levels (SOD, GSH-Px, MDA), intracellular calcium, and mitochondrial health	peptides increased PC12 cell viability under glutamate exposure, showing neuroprotection by activating antioxidant enzymes, inhibiting ROS production, reducing calcium influx, and regulating apoptotic proteins			activation of intracellular antioxidant enzymes (SOD and GSH-Px) via Keap1 inhibition, reducing ROS production, Ca^2+^ influx, and MMP collapse, while regulating apoptotic proteins Bax and Bcl-2
 ^77^			mice treated with walnut peptides were tested for seizure protection using PTZ, electroshock, and kindling models. The study also explored nitrergic and GABAergic pathway involvement	studies show that walnut peptides exhibit notable neuroprotective effects against PTZ-, MES-, and chemically induced kindling seizures in a dose-dependent manner	walnut peptides exert their neuroprotective effects primarily through GABA-A receptors
^41^	BV-2 cells treated with LPS and peptide were analyzed for viability, oxidative stress markers (ROS, SOD, CAT), cytokines, and protein expression by immunofluorescence and Western blot	WEKPPVSH showed antioxidant effects by reducing ROS and increasing SOD and CAT activity. It also exhibited anti-inflammatory activity by decreasing NO, TNF-α, IL-1β, IL-6, and the expression of iNOS and COX-2			the peptide’s protective mechanism involves activation of the Nrf2/HO-1 pathway, increasing Nrf2 and HO-1 expression, and inhibition of the NF-κB/p38 MAPK pathway, reducing p-p65, p-IκB-α, p-p38, and p65 nuclear translocation
^20^	HT-22 cells were cultured and treated with TW-7 prior to oxidative stress induction by H_2_O_2_. Viability was assessed by MTT, apoptosis and synaptic markers were analyzed by flow cytometry and immunofluorescence, and Western blot examined proteins involved in the cellular response	TW-7 showed neuroprotective effects in HT22 cells by increasing cell viability under oxidative stress and preserving synaptic plasticity			the peptide TW-7 inhibits MMP-9 expression via the NF-κB/iNOS pathway in bEnd.3 cells injured by Aβ 25–35 by binding to the fibronectin type II domain of MMP-9 and blocking its interaction with the substrate. This increases tight junction proteins, such as ZO-1 and Claudin-5, protecting blood-brain barrier (BBB) integrity
^21^	bEnd.3 cells were cultured and treated with TW-7 and Aβ 25–35 to assess neuroprotection. Cell viability (MTT), immunofluorescence, ROS, Western blot, and TEER assays were performed to evaluate blood-brain barrier integrity	the peptide TW-7 protected the blood-brain barrier in bEnd.3 cells injured by Aβ 25–35 by inhibiting MMP-9 expression and increasing ZO-1 and Claudin-5 proteins. TW-7 also reduced ROS production and restored BBB function	C57/N6 mice were given D-galactose to induce aging and treated with TW-7, vitamin C, or saline. After 8 weeks, they were evaluated using the Morris Water Maze (MWM), electron microscopy, immunofluorescence, and Western blot of the brain.	TW-7 treatment significantly improved learning and memory in mice with D-gal-induced dementia.	walnut peptides exert antioxidant and anti-inflammatory effects by boosting GSH and SOD, lowering MDA, 8-OHdG, and inflammatory cytokines, inhibiting AChE, and reducing iNOS, NO, and NF-κB activation, thereby modulating the Aβ/ROS/NF-κB pathway involved in Alzheimer’s disease
^48^			Kunming mice were induced with Alzheimer’s via Aβ25–35 and treated with walnut peptides. Memory tests and brain analyses for oxidative stress (SOD, GSH, MDA, AChE, NO) markers and proteins (iNOS, NF-κB) were performed	walnut peptides significantly improved memory and learning in mice with Aβ25–35-induced Alzheimer’s, as shown by MWM and Step-down tests	walnut peptides boost antioxidants (GSH, SOD), reduce oxidative stress markers (MDA, 8-OHdG), lower inflammatory cytokines (IL-6, IL-1β, TNF-α), inhibit AChE, decrease iNOS and NO, and suppress NF-κB activation, modulating the Aβ/ROS/NF-κB pathway key to Alzheimer’s progression
^22^	PC12 cells treated with peptides and glutamate were assessed for viability (MTT), cell damage (LDH), antioxidants (SOD, GSH-px, MDA), ROS, calcium, mitochondrial membrane potential, and apoptosis markers (Bax, Bcl-2) via Western blot, plus antioxidant activity by ABTS assay	the peptides GGW, VYY, and LLPF from WPHL protected PC12 cells treated with glutamate by reducing ROS production, Ca^2+^ influx, and mitochondrial membrane disruption. They also increased antioxidant protein activities (SOD and GSH-px)	rats were divided into five groups and treated with different substances during 5 days of sleep deprivation induced by multiple platforms. After behavioral tests, the hippocampus was isolated to measure antioxidant levels (SOD, GSH, GSH-px, CAT) and MDA	WPH and its fraction WPHL improved performance in sleep-deprived rats, especially WPHL. The enhancement in spatial memory was linked to reduced oxidative damage in the brain, with restored antioxidant enzymes and decreased MDA	the peptides GGW and VYY protected glutamate-treated PC12 cells by reducing antioxidant enzyme depletion (SOD, GSH-px) and ROS production. LLPF inhibited Ca^2+^ influx and prevented mitochondrial membrane potential loss. They also modulated apoptosis by lowering pro-apoptotic Bax and increasing anti-apoptotic Bcl-2 levels
^23^	BV-2 cells were treated with LPS to induce inflammation. Cell viability was assessed by MTT. Inflammatory proteins (iNOS, COX-2) were analyzed by Western blot, and TNF-α, IL-1β, and IL-6 gene expression by RT-PCR. ROS production was measured with DCFH-DA, and mitochondrial integrity by JC-1 fluorescence	the peptides LPF, GVYY, and APTLW reduced inflammation in BV-2 cells by lowering inflammatory mediators, enzymes (iNOS, COX-2), and cytokines. They also decreased ROS production and restored mitochondrial function, helping maintain redox balance	rats were divided into five groups and treated for 21 days with saline or peptides. After LPS-induced inflammation, memory was assessed using the Morris Water Maze. Brain analyses measured NO, PGE2, IL-1β, IL-6, TNF-α, antioxidant enzyme activities (SOD, GSH-px, CAT), and MDA levels	WPH and WPHL peptides improved memory in LPS-treated mice by reducing time and distance to find the platform, with WPHL more effective. They also lowered brain inflammatory mediators and cytokines and improved oxidative stress markers (SOD, CAT), while decreasing MDA levels	peptides inhibit iNOS and COX-2 expression, reducing inflammatory mediators (NO, PGE2) and cytokines (TNF-α, IL-1β, IL-6). They also lower ROS production and improve mitochondrial function by stabilizing its membrane. Hydrophobic and aromatic amino acids like Leu, Gly, and Trp enhance their anti-inflammatory action by aiding cell membrane interaction and signaling modulation
^19^	experiments were conducted in PC12 cells (neuronal model) exposed to 50 μM beta-amyloid peptide (Aβ25–35) for 24 h to induce oxidative stress and apoptosis	the peptides showed antioxidant effects by reducing ROS, increasing GSH-Px activity, raising ATP levels, and decreasing apoptosis. They also modulated the Akt/mTOR pathway, increasing p-Akt and p-mTOR, and promoted autophagy			the peptides reduce ROS, enhance GSH-Px, restore ATP levels, and promote autophagy by modulating Akt/mTOR signaling. Most peptides increase LC3-II, Beclin-1, LAMP1/2, and Cathepsin D, while KVPPLLY mainly reduces p62. Their effects are associated with hydrophobic and aromatic amino acids
^24^	conducted in HT-22 cells (immortalized murine neurons) treated with H_2_O_2_ to induce oxidative stress and mitochondrial damage	treatment increased expression of key mitophagy protein PINK1, enhanced antioxidant markers, restored redox balance, reduced ROS, protected against mitochondrial dysfunction	behavioral tests (Morris Water Maze) were performed in mice with scopolamine-induced cognitive impairment	significant improvement in learning and memory, preservation of mitochondrial integrity, and increased expression of mitophagy	the peptide stimulates PINK1-mediated mitophagy, removing damaged mitochondria and preventing cellular dysfunction. It also activates the NRF2/KEAP1/HO-1 antioxidant pathway, reducing oxidative stress
^62^	the effects of peptide PPKNW on the viability of HEK-293-E22G cells were determined by MTT assay, and Aβ aggregation was also evaluated	PPKNW showed no toxicity in HEK-293-E22G cells. Moreover, PPKNWespecially at a high dose, significantly reduced Aβ aggregation over 72 h, with more pronounced effects after 48 h	APP/PS1 mice were treated with peptide PPKNW for 12 weeks. After treatment, Morris Water Maze and shuttle box tests were conducted to assess cognitive function. Subsequently, immunohistochemistry was performed to detect Aβ42 plaques in the hippocampus, along with metabolomic analysis of blood samples and gene sequencing of fecal microbiota	PPKNW reduced amyloid plaque formation in the brain, significantly improved cognitive performance, increased serum levels of norepinephrine (NE) and isovalerate, and decreased levels of acetylcholinesterase (AChE) and valerate, demonstrating metabolic regulation favorable to neuronal function	reduction of β-amyloid aggregation and improvement of cognitive function through metabolic modulation (increased NE and decreased AChE), along with restoration of gut microbiota balance, which also influences neuroinflammatory and cognitive processes
^66^	SH-SY5Y cells were treated with the peptide SGGY and oxidative stress induced by H_2_O_2_. Cell viability (MTT), membrane integrity (LDH), ROS levels, mitochondrial membrane potential (MMP), antioxidant enzyme activities (SOD, CAT, MDA), apoptosis (flow cytometry), and protein expression (Western blot) were evaluated	reduction of intracellular ROS levels; preservation of mitochondrial membrane potential (MMP); inhibition of apoptosis and increased cell viability; restoration of endogenous antioxidant enzyme activities decrease in MDA content; promotion of Nrf2 nuclear translocation, inhibition of pro-apoptotic gene			SGGY reduces oxidative stress by neutralizing ROS and preserving mitochondrial membrane potential. It activates the Nrf2 pathway, stimulating the expression of antioxidant enzymes and strengthening cellular defense against oxidative stress. Additionally, it inhibits pro-apoptotic JNK/p38 MAPK pathways, preventing apoptosis and protecting neurons from mitochondrial dysfunction and cell death
^74^			mice with scopolamine-induced cognitive deficits were treated with WPH at low and high doses. Additional validation was performed in zebrafish using the same induction mode	improvement in behavior, restoration of cholinergic function, reduction of ROS, and increased expression of Nrf2, BDNF, and CREB. Neuroprotective effects in zebrafish	peptide treatment preserved memory by boosting ACh and AChR levels, inhibiting AChE, and upregulating BDNF, CREB, and Nrf2—effects likely linked to their low molecular weight and hydrophobicity
^10^			zebrafish model with groups exposed to scopolamine (model group), treated with Arg-containing peptides, and treated with walnut protein hydrolysate; behavioral tests were conducted followed by biochemical analysis of the brain	reduction in escape latency and total distance traveled to the target zone, along with an increase in entries into the target zone	arginine-containing peptides showed stronger neuroprotection than free arginine, with activity influenced by charge, arginine amount, peptide length, and cell penetration
^78^			mice were orally treated with a combination of D-galactose and aluminum chloride for 90 days. The treated group received the peptide daily by oral gavage	improved memory, hippocampal neuroprotection, reduced oxidative stress and inflammation, and positive modulation of the cholinergic system	the peptide exerts antioxidant effects, combating oxidative stress induced by d-gal and AlCl_3_, inhibiting pro-inflammatory cytokines and restoring cholinergic balance. This promotes neuronal protection and cognitive improvement. Its activity is probably linked to the high content of Trp, Asp, and Lys residues
^67^	PC12 cells were exposed to oxidative stress induced by H_2_O_2_. The cells were incubated with different concentrations of WP, and cell viability was analyzed using the MTT assay	treatment with H_2_O_2_ reduced cell viability to 57.2%. However, pretreatment with the peptide for 6 h increased viability to 76.9%, suggesting a neuroprotective effect against oxidative stress	zebrafish models were used to measure GSH levels, and mice underwent Morris water maze and step-through passive avoidance tests to assess learning and memory	moderate neuroprotection in zebrafish, mainly inhibiting extrinsic apoptosis caspases. In mice, it improved memory	the peptide modulates apoptosis and neurotrophic factors by decreasing Bax and increasing BDNF at low to medium doses, while high doses reverse these effects. It reduces GDNF at all doses, suggesting neuroprotection mainly at low to moderate levels
^26^	the study evaluated the inhibitory activity of synthetic peptides on purified human MAO-A and MAO-B enzymes	the peptides showed strong inhibitory activities against MAO-B			the peptides inhibit MAO-A and MAO-B enzymes, increasing brain levels of dopamine, serotonin, and norepinephrine, potentially improving mood and neuronal function
^25^	PC-12 cells were used to evaluate cell viability, oxidative stress, apoptosis, mitochondrial function, and stress-related protein expression	the peptides showed protective effects in PC-12 cells against H2O2-induced oxidative damage, enhancing cell viability and reducing reactive oxygen species (ROS) production and apoptosis			the mechanism involves activation of the SIRT3–FOXO3a pathway, enhancing mitochondrial function and reducing oxidative stress, with greater efficacy linked to a tryptophan-containing peptide
^72^			WKY and SHR rats received the peptide VHVV or ACE inhibitors for 24 weeks. Brain analyses were performed: immunohistochemistry, H&E staining, TUNEL assay, protein quantification (Lowry method), and Western blot	the peptide VHVV reversed memory deficits and neurodegeneration in hypertensive SHR rats	the peptides increased the expression of BDNF and activated neuroprotective pathways such as BDNF-TrkB, CaMKII-CREB, and PI3K-AKT-mTOR. These mechanisms promoted neuroplasticity, neuronal survival, and resistance to hypertension-mediated damage
^75^	to assess ROS, DPPH radical scavenging activity and Fe^3+^ reduction capacity were measured	peptides showed greater Al^3+^ chelation and antioxidant activity than regular soy protein, indicating potential to reduce Al^3+^ toxicity and oxidative stress	rats were treated with peptides and subjected to behavioral tests. Blood and tissue samples were then collected to assess aluminum (Al) levels, biochemical markers (AST, ALT, GSH, MDA), histopathology (HE), and stress-related proteins by Western blot	in mice exposed to Al^3+^, the peptides reduced aluminum accumulation, enhanced antioxidant defenses, prevented apoptosis, and regulated AMPK, alleviating brain and liver damage as well as cognitive deficits	the soybean peptide exerted neuroprotective effects by reducing oxidative stress, inhibiting apoptosis, and restoring AMPK activity
^28^	PC12 cells treated with lead acetate and peptides showed improved viability and antioxidant status, with modulation of Keap1, Nrf2, and TXNIP	BSPs alleviated lead-induced oxidative stress damage in PC12 cells			the peptides alleviated oxidative stress-related effects through the Keap1/Nrf2/TXNIP signaling pathway
^29^	HT22 cells were pretreated with the peptide exposed to Pb (20 μM) for 24 h. Cell viability was assessed at 450 nm. Total proteins were extracted, separated by SDS-PAGE, transferred to PVDF membranes, and probed with primary antibodies after blocking. Immunofluorescence assay was also performed	the peptide significantly reversed Pb-induced reduction in cell viability and alleviated neuroinflammation. Its protective effect is linked to activation of the AMPK/SIRT1 pathway and inhibition of mTOR signaling			the peptide suppressed BACE1 activity, reducing Aβ1–42 production and Tau hyperphosphorylation, thus alleviating Alzheimer’s pathology. Its antiamyloid and antineuroinflammatory effects may be mediated via modulation of the AMPK/SIRT1/NF-κB signaling pathway
^79^	effects of peptides on H_2_O_2_-induced oxidative damage in PC12 neuronal cells	the peptides, except those prepared with pepsin, showed a protective effect against oxidative stress-induced damage in PC12 cells, with the alcalase hydrolysate exhibiting the most pronounced protective effect			an antioxidant effect was observed along with a decrease in enzymes related to oxidative stress
^30^			lifespan, motility, α-synuclein aggregation, dopaminergic neurodegeneration, feeding behavior, oxidative stress (ROS and SOD), ATP levels, and gene expression (qRT-PCR) were assessed in *C. elegans*	increased lifespan, reduced α-synuclein aggregation, and protection of dopaminergic neurons against MPP^+^-induced neurotoxicity	reduces oxidative stress by increasing SOD activity and ATP levels; activates SKN-1, enhancing neuroprotection and resistance to neuronal degeneration
^31^			*C. elegans* were treated with the peptide and evaluated for motility, paralysis, polyQ aggregation, neuronal viability, oxidative stress, antioxidant activity, mitochondrial function, and RNAi-mediated molecular	the tests showed that the peptide increased nematode lifespan, alleviated polyQ-mediated toxicity, and reduced polyQ	the neuroprotective effects of PSC include reducing polyQ aggregation, decreasing oxidative stress, restoring mitochondrial function, and activating the SKN-1 signaling pathway
^16^			the study evaluated the effects of plant peptides on Aβ aggregation in *C. elegans*, assessing lifespan, motility, paralysis, oxidative stress, ROS production, and genetic responses via RT-PCR, Western blot, and microscopy	the peptide showed a protective effect against Aβ-induced toxicity in transgenic *C. elegans* and enhanced the nematodes’ ability to withstand oxidative stress	the results suggest that PSC-mediated protection against Aβ-induced toxicity and oxidative stress is mediated by SKN-1
 ^38^	HT-22 cells were treated with the peptide and exposed to NMDA and glycine, and cell viability was assessed using the CCK8 assay	the peptide prevented NMDA-induced damage and death in HT22 cells			
^37^	primary rat hippocampal neurons were exposed to NMDA-induced stress. Neuroprotection was evaluated by cell viability, mitochondrial potential, apoptosis, calcium levels, electrophysiology, protein expression, and caspase-3 activity	the peptide protected hippocampal neurons against NMDA-induced excitotoxicity			inhibits intracellular Ca^2+^ by modulating NMDA currents and regulating apoptotic proteins, dependent on NR2B-containing NMDA receptor inhibition
^55^	HUVEC and HT-22 cells used to assess YYLLVR toxicity; Ang II injury induced in HT-22; peptide’s protective effect tested pre- and postinjury; protein expression analyzed by Western blot	peptide improved vascular function and reduced inflammation in endothelial and neuronal cells by modulating RAS and increasing NO production, promoting neuroprotection			peptides showed potential to reduce α-synuclein aggregation and inhibit POP activity. Peptide modulates nitric oxide and renin-angiotensin systems, enhancing vasodilation (↑eNOS, NO), reducing inflammatory mediators (↓iNOS, COX-2, ET-1), and balancing RAS components (↓ACE, AGTR1; ↑AGTR2, ACE2, MAS1) to promote anti-inflammatory and neuroprotective effects
^17^	cell viability of SH-SY5Y after Aβ 1–42 damage by Alamar Blue; stress proteins by Western blot; ROS and mitochondrial membrane potential measured)	they demonstrated neuroprotective properties, evidenced by increased viability of SH-SY5Y cells subjected to Aβ1–42-induced oxidative damage			peptides reduced ROS and Aβ, restored mitochondrial potential, inhibited pro-apoptotic proteins Bax and Caspase-3, activated Akt/GSK-3β and Nrf2/HO-1 antioxidant pathways
^39^	antioxidant activity was tested using DPPH, ABTS, FRAP, and NOS assays	blue flower extracts of *Clitoria ternatea* showed higher antioxidant activity (93%) than white flowers, indicating greater potential against oxidative stress			peptides may inhibit amyloid-beta aggregation, preventing neurotoxic plaque formation, and also inhibit AChE, increasing acetylcholine levels and supporting neuronal communication
^34^	peptide fractions’ cytotoxicity and protective effects were tested in HMC3 cells. ROS, H2O2, NO, TNF-α, and IL-6 levels were measured, with microglial activation induced by LPS	fractions showed significant cytoprotective effects in HMC3 cells by reducing ROS, H2O2, NO, and pro-inflammatory cytokines (TNF-α, IL-6) after LPS stimulation			the neuroprotective mechanism involves reducing oxidative stress and modulating inflammation, mainly by inhibiting NF-κB pathways and decreasing inflammatory mediators and free radicals
^61^	peptide inhibitory activity was tested in SH-SY5Y cells treated for 72 h. α-synuclein aggregation was analyzed by immunofluorescence using a specific antibody and confocal microscopy	peptides showed potential to reduce α-synuclein aggregation and inhibit POP activity at the cellular level			the peptide acted as a mixed inhibitor of POP, while SPLGPA was a noncompetitive inhibitor. Both bound to POP via hydrogen bonds. Additionally, reduced α-synuclein aggregation in KCl-treated SH-SY5Y cells
^56^	BV-2 cells were treated with LPS and GPETAFLR. Cell viability (MTT), gene expression (qRT-PCR), microglia activation (FACS), and cytokine levels (ELISA) were analyzed	GPETAFLR was able to block LPS-induced inflammatory activation of microglia in BV-2 cells	mice were fed an HFD diet with or without GPETAFLR. After 8 weeks, brains were analyzed for cytokine expression (TNF-α, IL-1β, IL-6, and IL-10) and microglial cell profile	GPETAFLR decreased pro-inflammatory cytokines and increased IL-10 in high-fat diet mice, promoting microglial shift from M1 to M2 phenotype and improving microglial function	GPETAFLR inhibits pro-inflammatory microglial activation by reducing cytokines such as TNF-α, IL-1β, and IL-6. It also promotes microglial transition from M1 to M2, creating an anti-inflammatory environment that supports neuronal regeneration and CNS homeostasis
^33^	SH-SY5Y cells were pretreated with the peptide and then exposed to H_2_O_2_ (200 μM)	there was a 37% increase in cell viability, reduced LDH release, inhibition of ROS accumulation, maintenance of mitochondrial potential, regulation of Ca(2+); increased Bcl-2/Bax ratio; caspase-3 inhibition and reduced apoptosis			the Bcl-2/Bax ratio blocks cleavage of poly(ADP-ribose) polymerase by inhibiting caspase-3 activation, thereby reducing apoptosis; it also protects mitochondria and regulates intracellular Ca^2+^
 ^32^	model of neurotoxicity induced by 6-hydroxydopamine (6-OHDA) in SH-SY5Y cells (human neuroblastoma)	the peptide showed antioxidant activity measured by the FRAP assay; reduced reactive oxygen species (ROS) levels; restored mitochondrial dysfunction caused by 6-OHDA; and decreased LDH activity, indicating reduced membrane integrity loss.			antioxidant action, reduced oxidative stress and ROS levels, protection of mitochondrial function; increased BDNF as a compensatory response to BDNF decrease in SH-SY5Y cells after 6-OHDA exposure; and decreased LDH activity at the lowest concentration
^80^			DdY mice were used in the step-through passive avoidance test	R-6 enhanced memory consolidation at low doses, involving opioid receptors, as shown by naltrindole blockade	acts via selective activation of opioid receptors (mainly delta receptors), promoting memory consolidation. The peptide resists peptidase degradation due to an N-terminal YP sequence, allowing intestinal absorption, blood-brain barrier crossing, and direct central nervous system action
^36^	SK-N-SH cells were treated with the peptide and exposed to H_2_O_2_ to induce oxidative stress. Cell viability was assessed by MTT assay. Gene expression was measured by qPCR normalized to β-actin. Western blot analyzed BDNF and pERK levels, and immunostaining visualized BDNF in brain samples	in SK-N-SH cells, preincubation with FRP reduced H_2_O_2_-induced cell death by upregulating BDNF, NGF, and the antioxidant enzyme GST, helping protect cells from oxidative damage	male C57BL/6 mice were divided into 4 groups and treated via oral gavage and injection. After 24 h, they received scopolamine and underwent a passive avoidance test. Serum and hippocampus were collected to analyze ACh, AChE, SOD, NO, and MDA levels	the peptide increased BDNF levels in the hippocampus of mice and prevented scopolamine-induced cognitive deficits, improving memory and hippocampal function. The treatment also inhibited scopolamine’s negative effects on cholinergic neurotransmission, including AChE activity and ChAT regulation	it promoted neuroprotection by increasing BDNF levels and activating ERK/CREB/BDNF signaling, supporting neuronal survival and synaptic plasticity. It also regulates the cholinergic system by reducing AChE activity and activating ChAT, maintaining acetylcholine levels and aiding mAChR signaling, important for memory. Additionally, it decreases brain oxidative stress by boosting antioxidant activity like GST
^35^	assays were conducted on PC12 cells exposed to hydrogen peroxide-induced oxidative stress to evaluate gene expression modulation (Nrf2-Keap1/HO-1), endogenous antioxidant activity, and protective cellular effects	the treatment partially reversed cell damage and apoptosis, reduced LDH release and ROS levels, increased antioxidant enzyme activity, and boosted Nrf2 and HO-1 expression in oxidatively stressed cells	zebrafish model with scopolamine-induced cognitive deficit	improved memory and cognition; reduced brain AChE activity; lower oxidative stress and inflammation; increased expression of neuroprotective genes Bdnf, Nrf2, and Erg1	direct and indirect antioxidant action through activation of the Nrf2-Keap1/HO-1 pathway; reduction of ACh activity; positive modulation of neurotrophic factors and genes involved in synaptic plasticity. These effects are attributed to the peptides’ composition, rich in hydrophobic and aromatic amino acids

The selected articles provide a comprehensive basis for analysis, encompassing different peptide sequences, plant sources, and extraction and purification methodologies. The diversity of experimental approaches, both computational and laboratory-based, allows for an in-depth exploration of the potential neuroprotective effects of these peptides, although the underlying mechanisms remain poorly understood. The inclusion of the assessment of safety profiles contributes to the discussion of the clinical feasibility and future development of these compounds as neuroprotective agents.

### 3.2 Bibliometrics

Most of the analyzed studies were published between 2016 and 2025, with a marked increase observed especially from 2019 onward. The period from 2021 to 2024 accounted for the highest number of publications, indicating continued growth in interest in the topic (Figure 2A). China stands out in scientific output, contributing the majority of the studies (90%), while the remaining articles were distributed across various countries. This trend reflects both a sustained interest in the field and significant investment in research by Chinese institutions.

**Figure 2:**
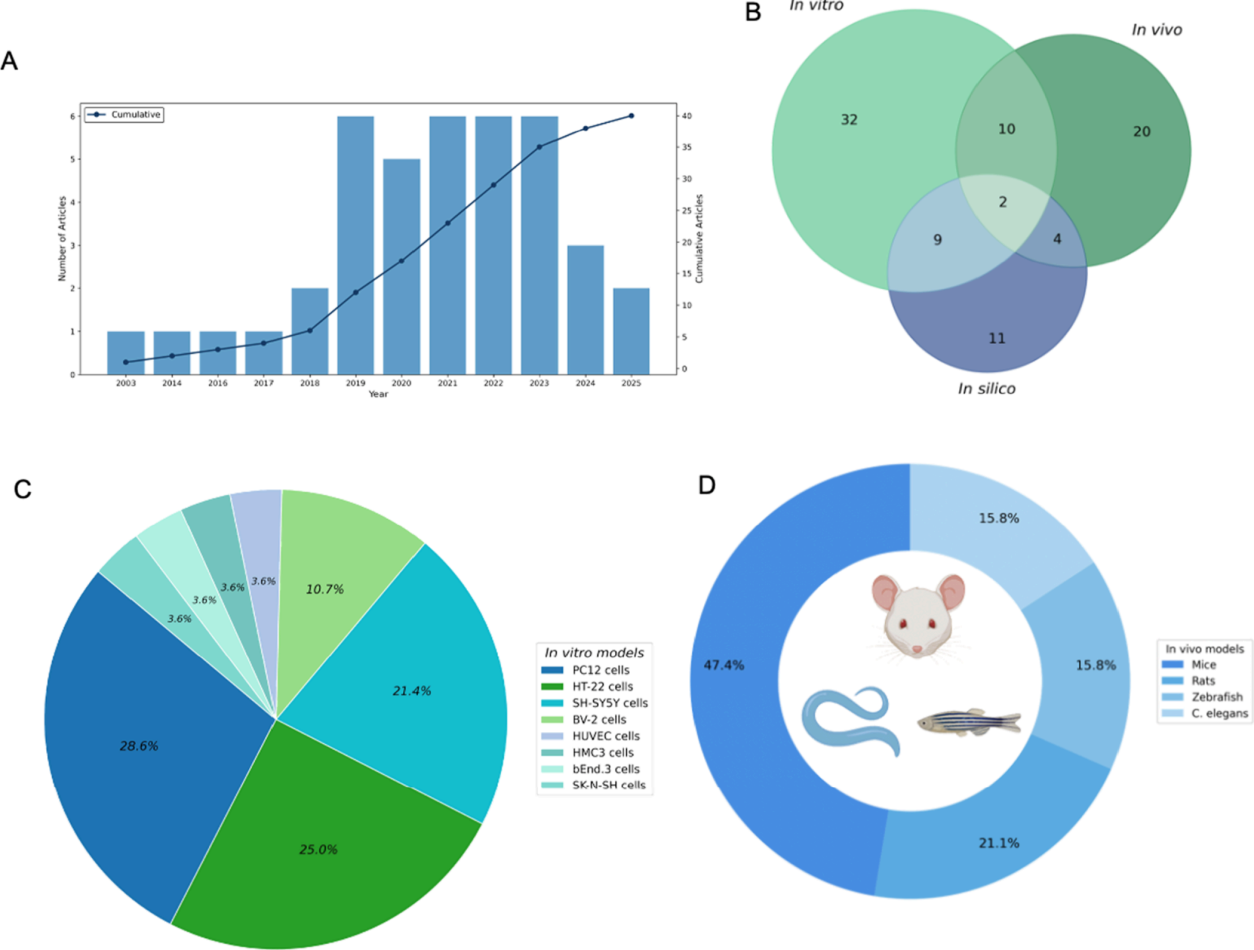
Bibliometric and methodological data of studies on plant-derived peptides and their effects on the pathophysiological mechanisms of neurodegenerative diseases. (A) Absolute and cumulative number of articles over the years. (B) Distribution of studies according to the experimental models used, with a Venn diagram showing the overlap among in vitro, in vivo, and in silico approaches. (C) Frequency of in vitro models employed in the selected studies. (D) Distribution of animal models used in the in vivo studies.

Regarding the experimental models employed, 32 studies used in vitro models (78.0%), 20 utilized in vivo models (48.8%), and 11 employed in silico approaches (26.8%). Most studies combined more than one type of model; however, only 2 studies (4.9%) integrated all three approaches simultaneously (Figure 2B).

Among the in vitro studies (n = 32), the most commonly used cell lines were PC12 (28.6%; *n* = 9), HT-22 (25.0%; *n* = 8), and SH-SY5Y (21.4%; *n* = 7). In the in vivo studies (*n* = 20), the most frequently used organisms were mice (47.4%; *n* = 9), rats (21.1%; *n* = 4), *C. elegans* (15.8%; *n* = 3), and zebrafish (15.8%; *n* = 3) (Figure 2C,D).

Among the analyzed articles, the main neuroprotective mechanisms investigated were oxidative stress (53.7%), followed by neuromodulation (26.8%) and neuroinflammation (24.4%). Other mechanisms explored included neuroplasticity (19.5%), apoptosis (19.5%), toxic protein aggregation (17.1%), and ion channel modulation (12.2%). These findings indicate that the predominant research focus is on combating oxidative stress, recognized as a key trigger of neurodegeneration, and may be interconnected with other identified mechanisms (Figure 3).

**Figure 3:**
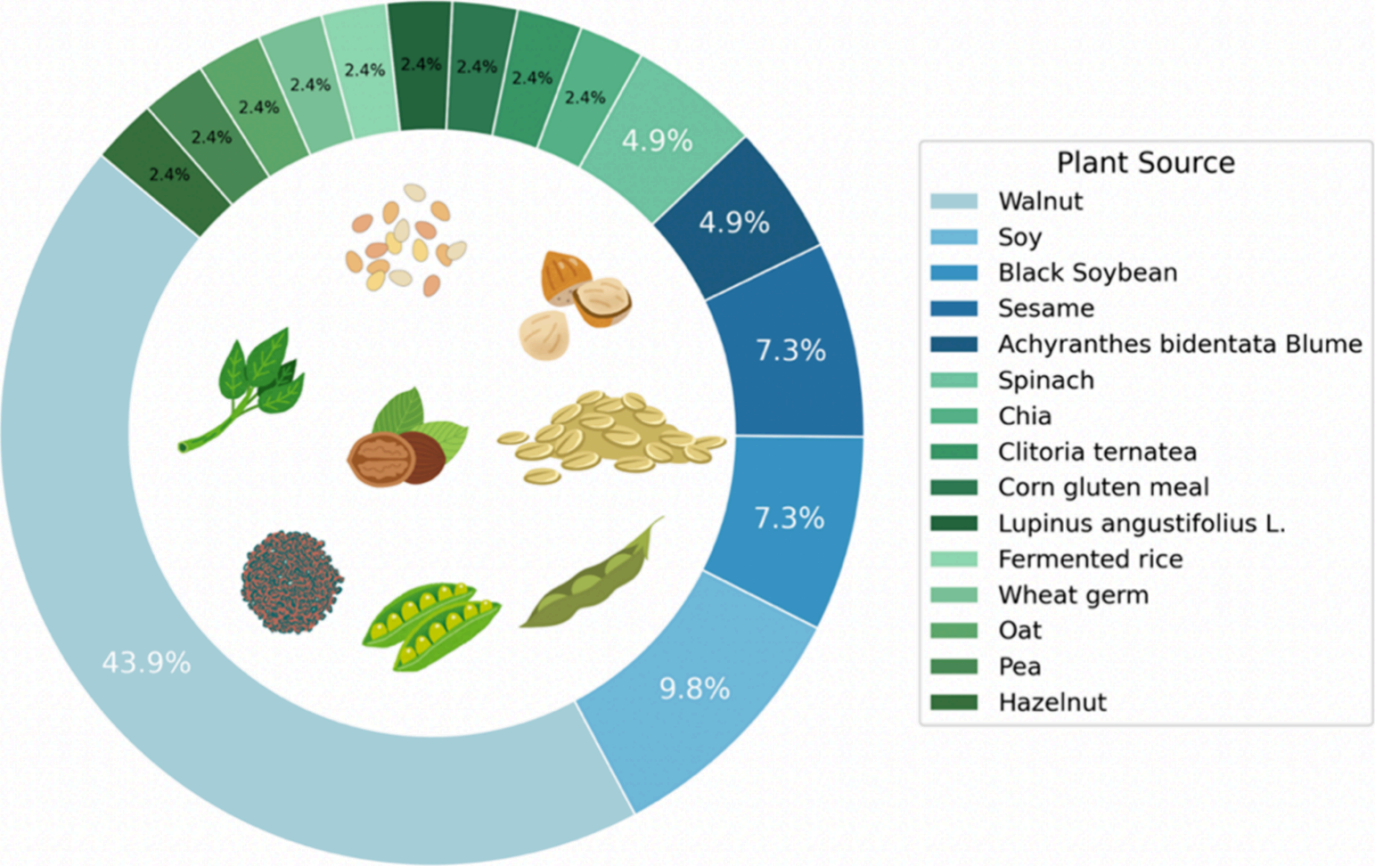
Plant sources used for the extraction of bioactive peptides with neuroprotective potential.

These results highlight the predominance of in vitro models in the investigation of the neuroprotective activity of plant-derived peptides while also emphasizing the complementary use of in vivo and in silico models, which allow for a more comprehensive and integrated assessment of biological effects and underlying pathophysiological mechanisms. Nevertheless, despite the considerable proportion of in vivo studies, further research using animal models is required to consolidate the validation of neuroprotective effects in more complex biological systems and to deepen the understanding of mechanisms of action in physiologically relevant contexts.

### 3.3 Plant-Derived Neuroprotective Peptides

Various plant sources have been investigated for their ability to generate peptides with beneficial effects in different cellular and animal models, revealing a trend reflected by the predominance of certain species throughout the analyzed studies (Figure 3).

Among the most investigated plant sources, walnuts (*Juglans regia*) have stood out for providing various bioactive peptides with potential neuroprotective effects. Among these, the peptide TWLPLPR was evaluated in primary hippocampal neurons subjected to toxicity induced by the Aβ_(25–35)_ fragment, a widely used β-amyloid model of AD. Treatment with TWLPLPR reduced neuronal hyperexcitability associated with Aβ exposure. In addition, application of the same peptide to PC12 cells previously treated with Aβ_(25–35)_ resulted in a significant decrease in oxidative stress and apoptosis levels.^18,19^

Additionally, the effects of TWLPLPR were assessed in different experimental models, including HT22 cells (murine hippocampal neurons), bEnd.3 cells (mouse brain endothelial cells), and C57BL/6 mice. In these models, treatment with the peptide resulted in significant improvements in cell viability as well as in cognitive and behavioral performance in vivo. Notably, peptide-treated mice exhibited enhanced memory and learning abilities, as evidenced by improved performance in the Morris water maze (MWM), further supporting the neuroprotective potential of TWLPLPR against diverse neurological insults.^20,21^

Other bioactive peptides extracted from walnuts include GGW, VYY, and LLPF, as rwell as GVYY, LPF, and APTLW. These peptides were investigated in PC12 and BV2 cell lines, respectively, to assess their efficacy in maintaining cell viability under exposure to various neurotoxic agents. The results demonstrated that all compounds were able to preserve cellular integrity, although their performance varied across the tested models, showing a generally favorable trend toward protection against oxidative and inflammatory stress. Notably, GGW, VYY, and LLPF exhibited a significant increase in ABTS radical scavenging activity, while GVYY, LPF, and APTLW were associated with modulation of antioxidant enzymes, including superoxide dismutase (SOD), catalase (CAT), and glutathione peroxidase (GSH-Px), indicating distinct cellular defense mechanisms against oxidative stress.^22,23^

Additionally, the peptide YVLLPSPK was analyzed using PC12 and HT22 cell lines as well as in mice, with significant neuroprotective effects observed. Key findings included reduced levels of oxidative stress and improved performance in learning and memory tests, suggesting a promising therapeutic potential for this peptide and walnuts as a source.^19,24^

Soy (*Glycine max*) also stands out among the most investigated plant sources for obtaining bioactive peptides with potential neuroprotective activity. The peptide SWGEDWGEIW was evaluated in PC12 cells, with analyses of cell viability, reactive oxygen species (ROS) production, and apoptosis, showing a significant improvement in cell survival.^23^ Similarly, the peptides YSPYPQ and PLYSN were investigated, and both promoted inhibition of the MAO-A and MAO-B enzymes, which are responsible for the degradation of monoaminergic neurotransmitters in the central nervous system, further supporting their potential as therapeutic targets in neurodegenerative diseases.^26,27^

Additionally, black soybean (*Glycine max* var. *nigrum*) has been used as a source of bioactive peptides, which demonstrated the ability to attenuate oxidative stress and neuroinflammation induced by lead (Pb^2+^) in PC12 and HT22 cells, respectively. These findings broaden the potential applications of these compounds against different types of neurotoxic insults, reinforcing their potential as neuroprotective agents.^28,29^

Peptides extracted from peas also showed promising results. Compounds such as NF, GF, and RP exhibited positive effects in SH-SY5Y cells exposed to aggregates such as Aβ_1_–_4__2_, indicating relevant cytoprotective properties.^17^ Sources such as sesame, including preparations like sesame cake, were also investigated for their neuroprotective potential, showing antioxidant effects, protection of dopaminergic neurons, and reduction of toxic compound aggregation in *Caenorhabditis elegans*.^30,31^

Although less frequently reported in the literature, other plant sources such as spinach (*Spinacia oleracea*)^32^ wheat germ (*Triticum aestivum*),^33^ chia (*Salvia hispanica*)^34^ oats (*Avena sativa*),^35^ and fermented rice (*Oryza sativa*)^36^ have been explored as matrices for bioactive peptide production. These compounds demonstrated antioxidant activity and neuroprotective effects in various experimental models, including cell lines such as SK-N-SH, PC12, SH-SY5Y, and HMC3, as well as in vivo mouse models. Although less conventional, these sources increase the structural diversity of the peptides studied, which may facilitate the identification of new candidates with therapeutic potential in neurodegenerative diseases.

Some studies have also explored plant species traditionally used in folk medicine as promising sources of bioactive peptides with therapeutic potential. One example is *Achyranthes bidentata* Blume, known as Chinese ox knee, from which the peptide bidentatide was isolated. This compound demonstrated significant neuroprotective effects in both rats and HT22 cells, promoting hippocampal neuron protection and preventing cell death.^37,38^ Similarly, *Clitoria ternatea* has been recently investigated, and studies have identified peptides present in the plant’s blue flower exhibiting neuroprotective activity in cellular models.^39^ The phosphate buffer extract of the blue flower also showed high ABTS radical scavenging activity, highlighting its antioxidant potential. The use of these plant species as matrices for bioactive compound extraction broadens the scope of research on natural therapies aimed at the prevention and treatment of neurodegenerative diseases.

Considering this landscape, the combination of different experimental models and plant sources reinforces the potential of peptides as neuroprotective agents in diverse contexts of neuronal damage. The integrated use of cell lines and animal models strengthens the validity of the findings and facilitates their extrapolation to more complex pathophysiological scenarios. At the same time, the structural diversity of peptides identified from various plant matrices expands therapeutic possibilities, contributing to the discovery of new compounds capable of modulating key processes involved in neurodegenerative diseases.

### 3.4 Extraction, Purification, and Identification Techniques

The production of bioactive peptides from plant sources involves an integrated approach combining enzymatic, chromatographic, and spectrometric techniques. Functional peptides derived from food proteins can be obtained through various processes, particularly enzymatic hydrolysis and fermentation.^13^ Enzymatic hydrolysis has emerged as a fundamental step for generating peptide fragments, as it involves the controlled cleavage of peptide bonds by specific proteolytic enzymes, leading to the release of smaller peptides with enhanced biological activity.^12^ This process is carried out using different enzymes depending on the specificity of the protein matrix. For example, Flavourzyme has been applied to pea protein extracts,^17^ whereas a combination of pepsin and pancreatin has been used for chia seeds.^34^ In soybean protein, collagenase has been employed for peptide production.^26^

Complementarily, extraction procedures using solvents and buffers, such as acetate buffer,^30^ acetonitrile, and methanol,^25^ facilitated protein solubilization and subsequent peptide release. Physical methods, including centrifugation and filtration, were also widely employed to clarify samples and remove particulate residues.^36^ Notably, in studies with walnuts, simulated gastrointestinal digestion was used as an alternative to generate bioactive peptide fractions in a manner more physiologically relevant to humans.^10^

For peptide purification, gel filtration chromatography was widely used due to its ability to fractionate compounds based on molecular weight.^38^ Examples include the use of Sephadex G-25 and G-15 columns in the separation of walnut peptides,^22,41^ as well as in soybean extracts.^25,26^ Ion-exchange chromatography was also frequently employed, particularly for matrices such as sesame and sesame cake, due to its efficiency in separating peptides with different electrical charges.^40^

High-performance liquid chromatography (HPLC) played a prominent role, serving as both a purification and identification method due to its high resolution and sensitivity. Ultrafiltration was also widely applied, either alone or in combination with chromatographic methods, promoting selective and efficient fractionation based on molecular size. This combined approach was particularly effective in studies involving walnut and soybean proteins,^42,29^ optimizing the isolation of fractions with potential bioactivity.

Peptide identification predominantly relied on mass spectrometry (MS) techniques, which are recognized for their high sensitivity, accuracy, and sequencing capability. LC-MS/MS (liquid chromatography coupled with tandem mass spectrometry) was widely used across studies, such as in the identification of peptides derived from fermented rice using the MALDI-TOF/TOF-MS technique (Matrix-Assisted Laser Desorption/Ionization – Time of Flight/Time of Flight).^36^ Techniques such as UPLC-QTOF-MS/MS with electrospray ionization (ESI) were also applied for the identification of neuroprotective peptides obtained from walnuts,^22^ while Q-TOF-MS was employed for the analysis of peptides extracted from *A. bidentata* Blume.^37^ For sesame cake, MS Q-Exactive was utilized, and the HPLC-ESI-MS/MS technique demonstrated broad applicability for both purification and characterization of peptides,^31^ standing out for its versatility and analytical efficiency.

Moreover, the UHPLC-QE-MS technique was successfully applied for the identification of soybean-derived peptides, providing excellent resolution, accuracy, and the capability to analyze complex mixtures.^25^ Thus, the strategic combination of these techniques enabled not only the extraction and isolation of bioactive peptides but also their precise identification, significantly contributing to the evaluation of their therapeutic potential.

### 3.5 Mechanisms of Action of Neuroprotective Peptides

#### 3.5.1 Antioxidant Activity and Modulation of Oxidative Stress

Oxidative stress is one of the central mechanisms involved in the onset and progression of neurodegenerative diseases. Recent studies have demonstrated that plant-derived peptides exhibit significant antioxidant activity and are able to modulate endogenous cellular defense systems and protect neuronal cells from damage induced by ROS. These peptides can attenuate oxidative stress through multiple complementary mechanisms, including the direct scavenging of free radicals, chelation of pro-oxidant metal ions, and enhancement of endogenous antioxidant defenses. Together, these effects contribute to reducing oxidative damage and preserving neuronal integrity.^24,43–45^

Peptides derived from walnuts have been extensively studied due to their antioxidant potential. These peptides have been shown to mitigate oxidative stress in SH-SY5Y cells.^43^ Similarly, peptides extracted from walnuts attenuate oxidative stress in PC12 cells through the activation of intracellular antioxidant enzymes, such as SOD and GSH-PX.^10^ This effect is mediated by the inhibition of the Keap1–Nrf2 pathway, which has been the most frequently identified pathway in the literature linking oxidative stress with neuroprotective peptides. Inhibition of Keap1 promotes the nuclear translocation of the transcription factor NRF2, which induces the expression of antioxidant proteins responsible for protecting cells against oxidative damage.^46^

Additionally, it was demonstrated that, besides activating NRF2, walnut peptides induce increased expression of the enzyme heme oxygenase-1 (HO-1), thereby enhancing the antioxidant response in HT-22 cells (mouse hippocampal cells).^24^ HO-1 degrades the heme group into free iron, carbon monoxide, and biliverdin, the latter subsequently converted into bilirubin, a potent cellular antioxidant.^47,48^ Moreover, studies indicate that walnut peptides reduce oxidative stress biomarkers, such as malondialdehyde (MDA) and 8-hydroxy-2’-deoxyguanosine (8-OHdG), increase intracellular levels of GSH and SOD, and prevent the depletion of antioxidant enzymes SOD and GSH-Px.^22,48^

Sesame-derived peptides also demonstrate significant antioxidant activity, evidenced by increased SOD enzyme activity and improved mitochondrial function, including elevated ATP production. In vitro assays showed that these peptides neutralize free radicals in a dose-dependent manner, with DPPH radical scavenging rates reaching nearly 100% at 1.8 mg/mL.^14^ In *C. elegans* models, activation of the transcription factor SKN-1, a functional homologue of mammalian NRF2, was observed and associated with the regulation of ROS production.^14,49^

In parallel, increased activities of CAT, SOD, GPx, and glutathione reductase (GR) were reported in PC12 cells treated with black-soybean-derived peptides, an effect attributed to inhibition of the Keap1/Nrf2/TXNIP pathway.^28^ Suppression of thioredoxin-interacting protein (TXNIP) expression enhances cellular antioxidant activity. Complementing these findings, soybean peptides were shown to activate the SIRT3–FOXO3a pathway in PC12 cells, in which deacetylation of FOXO3a by the mitochondrial sirtuin SIRT3 promotes mitochondrial protection against oxidative stress and induces the expression of essential antioxidant genes, thereby maintaining mitochondrial homeostasis.^25,49,50^

#### 3.5.2 Modulation of Neuroinflammation: Interaction with Pro-Inflammatory Cytokines and Cellular Signaling Pathways

Neuroinflammation is an inflammatory response within the brain or spinal cord, primarily orchestrated by glial cells, especially microglia and astrocytes, which act as immune sensors and effectors in the central nervous system. This response is mediated by the production of key pro-inflammatory cytokines, such as interleukin-1 beta (IL-1β), interleukin-6 (IL-6), and tumor necrosis factor-alpha (TNF-α), as well as chemokines, secondary messengers like nitric oxide (NO) and prostaglandins, and ROS.^51,52^

Although these glial cells play an essential role in brain defense by removing pathogens and cellular debris, prolonged activation can result in a neurotoxic environment, characterized by oxidative stress, mitochondrial dysfunction, and neuronal death.^53,54^ This chronic inflammatory state has been widely associated with the progression of neurodegenerative diseases, such as AD, PD, and amyotrophic lateral sclerosis (ALS), making the modulation of neuroinflammation a relevant and promising therapeutic target.^55^

Consequently, several recent studies have investigated the ability of bioactive peptides to attenuate neuroinflammation through different molecular pathways. One study demonstrated that the peptide YYLLVR, isolated from hazelnut, is capable of reducing inflammatory processes in HT-22 neuronal cells through modulation of the nitric oxide (NO) and renin–angiotensin (RAAS) systems.^55^ Treatment with this peptide promoted vasodilation by increasing endothelial nitric oxide synthase (eNOS) expression and NO production, while simultaneously reducing the expression of inflammatory mediators such as inducible nitric oxide synthase (iNOS), cyclooxygenase-2 (COX-2), and endothelin-1 (ET-1).^55^

Peptides isolated from walnuts (*J. regia*) were evaluated in HMC3 microglial cells stimulated with lipopolysaccharide (LPS), with reductions in TNF-α, IL-1β, IL-6, and NO levels, as well as iNOS and COX-2 expression.^41^ These effects were mainly attributed to inhibition of the NF-κB/p38 MAPK signaling pathway, particularly through suppression of the nuclear translocation of the NF-κB p65 subunit, a key factor in the transcription of pro-inflammatory genes. Complementarily, the TW-7 peptide, also derived from walnuts, inhibits MMP-9 expression via NF-κB/iNOS in bEnd.3 endothelial cells injured by Aβ 25–35.^21^ The peptide binds to the type II fibronectin domain of MMP-9, blocking its interaction with the substrate. This action increased tight junction proteins, such as ZO-1 and Claudin-5, protecting blood–brain barrier (BBB) integrity and providing an additional mechanism of neuroprotection and neuroinflammation control.^21^

Additionally, anti-inflammatory effects were reported in HMC3 microglial cells treated with peptides derived from *Salvia hispanica* (chia), observing a significant reduction in LPS-induced TNF-α and IL-6 levels.^34^ Anti-inflammatory action of walnut-derived peptides was also described, with reductions in NO and prostaglandin E_2_ (PGE_2_) production, as well as TNF-α, IL-1β, and IL-6 levels.^23^ The relevance of hydrophobic and aromatic residues, such as leucine (Leu), glycine (Gly), and tryptophan (Trp), which enhance peptide interactions with the cell membrane and positively influence intracellular signaling, was also highlighted.

Finally, the peptide GPETAFLR, extracted from *Lupinus angustifolius*, was analyzed in BV2 cells and a murine model of LPS-induced inflammation.^56^ Treatment significantly reduced TNF-α, IL-1β, and IL-6 expression and induced microglial phenotype switching from M1 (pro-inflammatory) to M2 (anti-inflammatory), contributing to a more favorable cerebral environment for neuroprotection. These findings reinforce the role of bioactive peptides as promising tools for mitigating neuroinflammation associated with neurodegenerative diseases such as AD, PD, and ALS.

#### 3.5.3 Inhibition of Neurotoxic Protein Aggregation

A common histopathological feature of neurodegenerative diseases is the formation of protein aggregates, which may occur due to mutations that render disease-specific proteins prone to aggregation or increase their intracellular concentration.^57^ The main proteins implicated in the formation of these aggregates include, among others, β-amyloid, tau, and α-synuclein. Over time, all of these disease-associated proteins promote synaptic dysfunction and loss, ultimately leading to neuronal cell death.^57^

In addition to synaptic dysfunction, other cellular alterations common to major neurodegenerative diseases include calcium signaling abnormalities, mitochondrial dysfunction, oxidative stress, and neuroinflammation. In other words, protein aggregates can amplify these harmful cellular processes, creating a vicious cycle that exacerbates neural degeneration and disease progression.^58,59^

Considering the central role of protein aggregates in neurodegeneration, several studies have evaluated the effect of natural peptides on modulating these processes. Peptides derived from pea were investigated in SH-SY5Y cells, showing a reduction in β-amyloid protein levels as well as a decrease in oxidative stress induced by exposure to the Aβ1–42 peptide.^17^ Similarly, peptides extracted from sesame demonstrated neuroprotective potential by reducing the level of aggregation of polyglutamine (polyQ) proteins in *C. elegans*. These polyQ repeats arise from pathological expansions of the CAG trinucleotide in the coding region of specific genes and, when translated, form aggregates in neuronal cells, contributing to degenerative processes.^60^

Furthermore, several studies have investigated the impact of bioactive peptides on the aggregation of proteins such as α-synuclein, which is involved in PD pathophysiology. Peptides derived from sesame cake were able to reduce α-synuclein levels in *C. elegans* models.^30^ Similarly, peptides obtained from maize gluten flour were effective in reducing α-synuclein aggregates in SH-SY5Y cells.^61^ Walnut-derived peptides were also reported to significantly reduce β-amyloid formation in a transgenic APP/PS1 murine model, reinforcing the potential of these compounds in preventing toxic protein aggregation.^62^

#### 3.5.4 Apoptosis

Apoptosis is a genetically programmed biochemical process responsible for the controlled removal of cells, playing essential roles under both physiological and pathological conditions.^59^ Physiologically, it acts as a homeostatic mechanism, regulating cell populations during the formation, maturation, and maintenance of systems.^64,65^ Pathologically, apoptosis is activated in response to cellular damage caused by diseases, stressors, or aging. The activation of proteases from the caspase family, as well as alterations in the expression of gene groups such as Bax and Bcl-2 (Bcl-2-associated X protein and B-cell lymphoma 2), plays a central role in apoptosis.^63^ In this context, the apoptotic process is fundamental to the pathophysiology of neurodegenerative diseases, in which the death of neuronal populations progressively compromises nervous tissue functions.

Consequently, several studies have sought to mitigate apoptotic and inflammatory damage characteristic of neurodegenerative diseases through the use of plant-derived peptides.^19,37,10,66^ The walnut-derived peptide SGGY was employed in human SH-SY5Y neuroblastoma cells exposed to oxidative stress induced by hydrogen peroxide (H_2_O_2_). This peptide reduced ROS and MDA levels, maintained mitochondrial membrane potential, restored the activity of antioxidant enzymes, and modulated the expression of apoptosis-related genes, including the kinases p38 and JNK (c-Jun N-terminal kinase).^66^ The accumulation of reactive species and mitochondrial membrane potential imbalance serves as a trigger for the initiation of the apoptotic process.

As previously mentioned, certain gene or protein groups act as key regulators of apoptosis. Among them are Bax and Bcl-2 (members of the Bcl-2 family), and caspases.^66^ Bax is a pro-apoptotic protein, whereas Bcl-2 is antiapoptotic.^33,66^ The balance between these two proteins functions as a “switch” for the apoptotic process.^33^ In a study, walnut peptides (*J. regia* L.) were applied to PC12 cells exposed to H2O2-induced oxidative stress and to zebrafish models to evaluate their neuroprotective activity.^67^ The results demonstrated inhibition of caspases 3, 7, and 8, reduction of Bax and GDNF (glial cell line-derived neurotrophic factor) expression, and increased expression of BDNF (brain-derived neurotrophic factor).^67^ These findings suggest a neuroprotective effect of the peptide as well as attenuation of learning and memory deficits.

Similarly, the soy-derived peptide SWGEDWGEIW in PC12 cells exposed to H_2_O_2_-induced oxidative stress inhibited oxidative cellular damage, enhanced cellular respiration, and increased ATP production. Moreover, the results showed a dose-dependent inhibition of Bax and increased expression of Bcl-2. At a concentration of 100 μg/mL, the Bax/Bcl-2 ratio increased approximately 3.2-fold, suggesting neutralization of H_2_O_2_-induced neurotoxicity, maintenance of mitochondrial integrity, and inhibition of mitochondria-dependent apoptosis.^25^

Another relevant neuroprotective mechanism related to cell death was reported.^19^ In this study, treatment with walnut-derived peptides (*Juglans mandshurica* Maxim) WLPLPR, YVLLPSPK, and KVPPLLY prevented ROS production, enhanced GPx activity, increased ATP levels, and improved apoptotic conditions in PC12 cells exposed to Aβ(25–35). Immunofluorescence and Western blot analyses revealed that the tested peptides regulate the Akt/mTOR kinase signaling pathways through serine residues present in both enzymes. Furthermore, the peptides promoted autophagy by increasing the LC3-II/LC3-I and beclin-1 levels while reducing the level of p62 expression. Additionally, the peptides were able to elevate LAMP1, LAMP2, and cathepsin D levels, promoting autolysosome formation and, consequently, accelerating ROS clearance. Thus, the study suggests that the examined peptides modulate oxidative stress through the promotion of autophagy under the described conditions.^19^

#### 3.5.5 Ionic Modulation and Neuroplasticity

Multiple neuronal functions are regulated by ionic homeostasis and signaling, including synaptic activity, neuroplasticity, and cell survival.^68–70^ Calcium (Ca^2+^), for instance, plays a key role not only in physiological processes but also in cell death and alterations in signaling and metabolism observed in neurodegenerative diseases such as AD, PD, and HD.^70^ A positive feedback loop between the loss of Ca^2+^ homeostasis and the accumulation of proteins such as Aβ, α-synuclein, and huntingtin is characteristic of the pathophysiology of these disorders.^68,69^

In this context, a peptide derived from the flower of *A. bidentata* (bidentatide) was investigated in cultures of hippocampal neurons subjected to NMDA (*N*-methyl-d-aspartate) receptor-induced hyperexcitation to assess its neuroprotective effect. Pretreatment with bidentatide was able to prevent NMDA-induced apoptosis through mechanisms involving reduction of intracellular Ca^2+^, regulation of apoptotic proteins, and inhibition of ionic currents triggered by NMDA receptor activation.^37^

Similarly, peptides such as WSREEQEREE and ADIYTEEAGR, derived from walnuts, demonstrated neuroprotective effects in PC12 cells exposed to glutamate toxicity, primarily by reducing excessive Ca^2+^ influx induced by NMDA receptor activation. This modulation of intracellular calcium contributed to preventing mitochondrial dysfunction and cell damage associated with excitotoxicity.^10^

Furthermore, the peptide RVF, derived from wheat germ, was analyzed. Neuroprotection was observed, as RVF reduced prolonged intracellular calcium elevation induced by H_2_O_2_ in neurons. Treatment with RVF protected the integrity of cellular membranes and calcium pumps, contributing to the maintenance of ionic homeostasis and preventing mitochondrial dysfunction-related neuronal apoptosis.^33^

Among the nervous system functions affected by ionic homeostasis, neuroplasticity, or synaptic plasticity is particularly important for healthy mental functioning, defined as the ability of the nervous system to modify the strength of neuronal connections in response to experiences or damage.^70^ This complex property enables structural and functional reorganization of neurons, which is vital for learning, memory, and adaptation to the environment. This process occurs across multiple temporal and spatial scales,^70^ and peptides derived from fermented rice have shown neuroprotective potential and the ability to preserve neuronal plasticity. In a murine model of scopolamine-induced memory deficit, increases were observed in biomarkers related to synaptic plasticity and neuroprotection, such as BDNF (brain-derived neurotrophic factor), a key molecule for the development of neuronal circuits, neural regeneration, and maintenance of synaptic plasticity, and PSD95 (postsynaptic density protein 95), in addition to prevention of cognitive déficits.^36,71,72^ Similarly, administration of soy-derived peptides resulted in increased BDNF expression and activation of neuroprotective pathways, including BDNF-TrkB, CaMKII-CREB, and PI3K-AKT-mTOR, in rats.^75^

Additionally, the walnut-derived peptide TWLPLPR was investigated for its neuroprotective effects, emphasizing synaptic plasticity mediated by the classical complement system, specifically the C1q component, in HT22 cells subjected to H_2_O_2_-induced oxidative stress.^20^ Treatment reduced C1q expression and consequently complement C3, while increasing levels of synaptic proteins SYP (synaptophysin) and PSD95, contributing to the preservation of synaptic connections. The peptide also inhibited apoptosis and modulated the IP3/PLC/IP3R pathway, promoting intracellular calcium homeostasis and supporting synaptic plasticity.^20^

These findings reinforce the promising role of plant-derived peptides in regulating molecular mechanisms essential for maintaining synaptic integrity and neuronal functionality.

#### 3.5.6 Modulation of Cholinergic Neurotransmission via Acetylcholinesterase Inhibition

Acetylcholinesterase (AChE) is a serine hydrolase responsible for terminating neurotransmission at cholinergic synapses by rapidly hydrolyzing acetylcholine.^73^ This enzyme has been an important therapeutic target in AD, in which acetylcholine levels are reduced.^73^

In a recent study, it was demonstrated that peptides isolated from *C. ternatea* modulate cholinergic neurotransmission through AChE inhibition. Following purification and characterization, the peptides APCPNR, LGLFR, LIPQE (from the blue flower), and SISWSS (from the white flower) were subjected to molecular docking against AChE, revealing binding energies ranging from −12.03 to −13.95 kcal/mol.^39^ These values suggest that the peptides can stably bind to the enzyme’s active site, thereby reducing its activity, preserving synaptic acetylcholine levels, and potentially mitigating AD-related cognitive deficits.^39^

Similarly, it was shown that a protein hydrolysate from walnut (WPH) not only improved memory performance but also directly inhibited AChE in mouse and zebrafish models of scopolamine-induced memory impairment.^74^ WPH reduced AChE enzymatic activity by approximately 20–35%, restoring acetylcholine levels and improving behavioral responses. Moreover, the dipeptide FY, isolated from WPH, displayed high docking affinity with AChE (Vina = – 9.8 kcal/mol), reinforcing its potential role as an enzyme inhibitor and elucidating the cholinergic mechanism underlying its neuroprotective effect.^74^

Additionally, it was confirmed in a zebrafish model that peptides derived from oat protein hydrolysate (OPH) positively modulated cholinergic neurotransmission. Three sequences, DFVADHPFLF, HGQNFPIL, and RDFPITWPW, reversed scopolamine-induced memory deficits and significantly reduced the scopolamine-induced increase in AChE activity, with RDFPITWPW showing the strongest enzymatic inhibition among the candidates tested.^35^ These results indicate that oat-derived peptides may act as AChE inhibitors, elevating synaptic acetylcholine levels and promoting recovery of cognitive function under cholinergic dysfunction.^35^

Finally, a study demonstrated that pretreatment with fermented rice peptides attenuated the effects of scopolamine-induced acetylcholine reduction and AChE activity elevation. Furthermore, peptide administration prevented the decrease of choline acetyltransferase (ChAT) and muscarinic acetylcholine receptors 1 and 3 (mAChR1/3) in the hippocampus of mice (Figure 4).^36^

**Figure 4:**
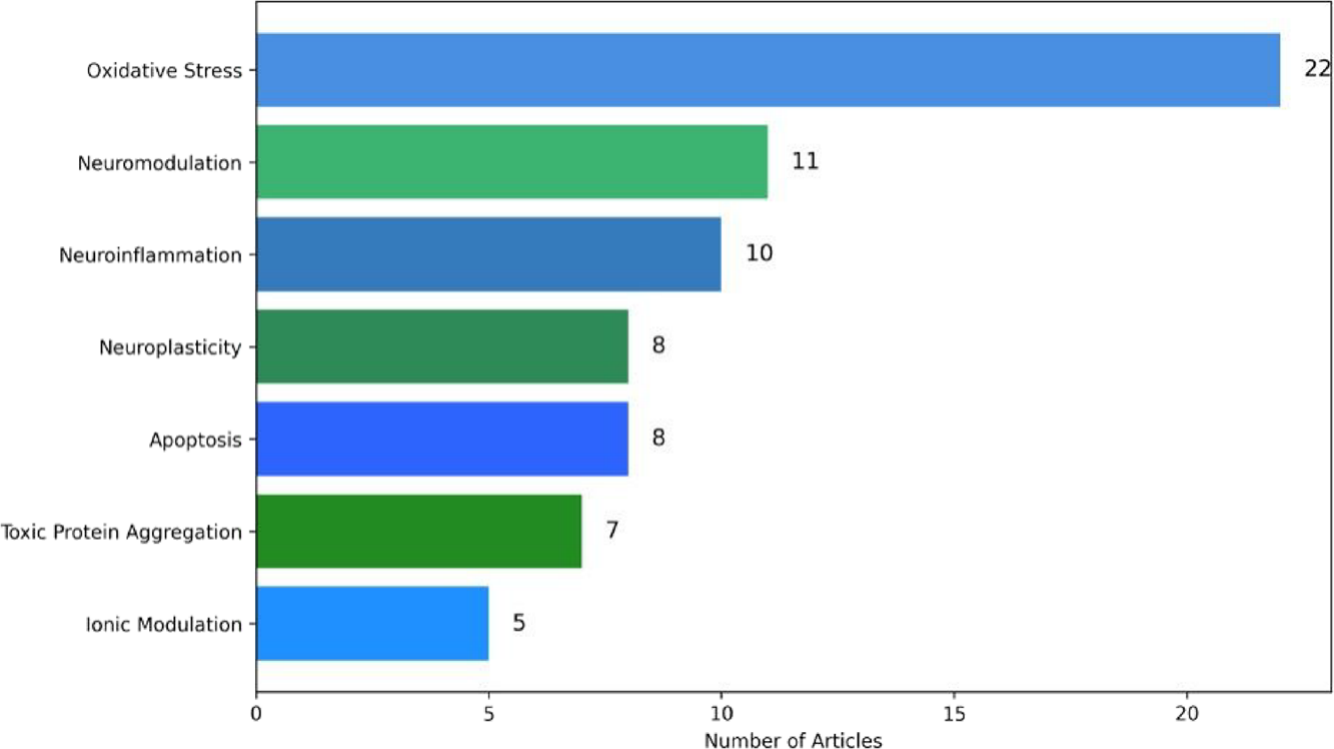
Main neuroprotective mechanisms investigated in the analyzed studies.

### 3.6 Modification of Plant-Derived Peptides to Enhance Neuroprotective Effects

Few studies have tested the effects of peptide modification on neuroprotective activities. It was demonstrated that walnut-derived peptides (WSREEQEREE and ADIYTEEAGR) have enhanced neuroprotective capacity in PC12 cells through the addition of an arginine residue to their sequence.^10^ This modification increases cell viability when exposed to glutamate. Arginine is a precursor to nitric oxide, which plays an important modulatory role in memory and learning.^10^ Therefore, this is the likely reason for the improved neuroprotective activity. A subsequent study confirmed this enhanced neuroprotective activity in zebrafish with scopolamine-induced amnesia (Figure 5).

**Figure 5:**
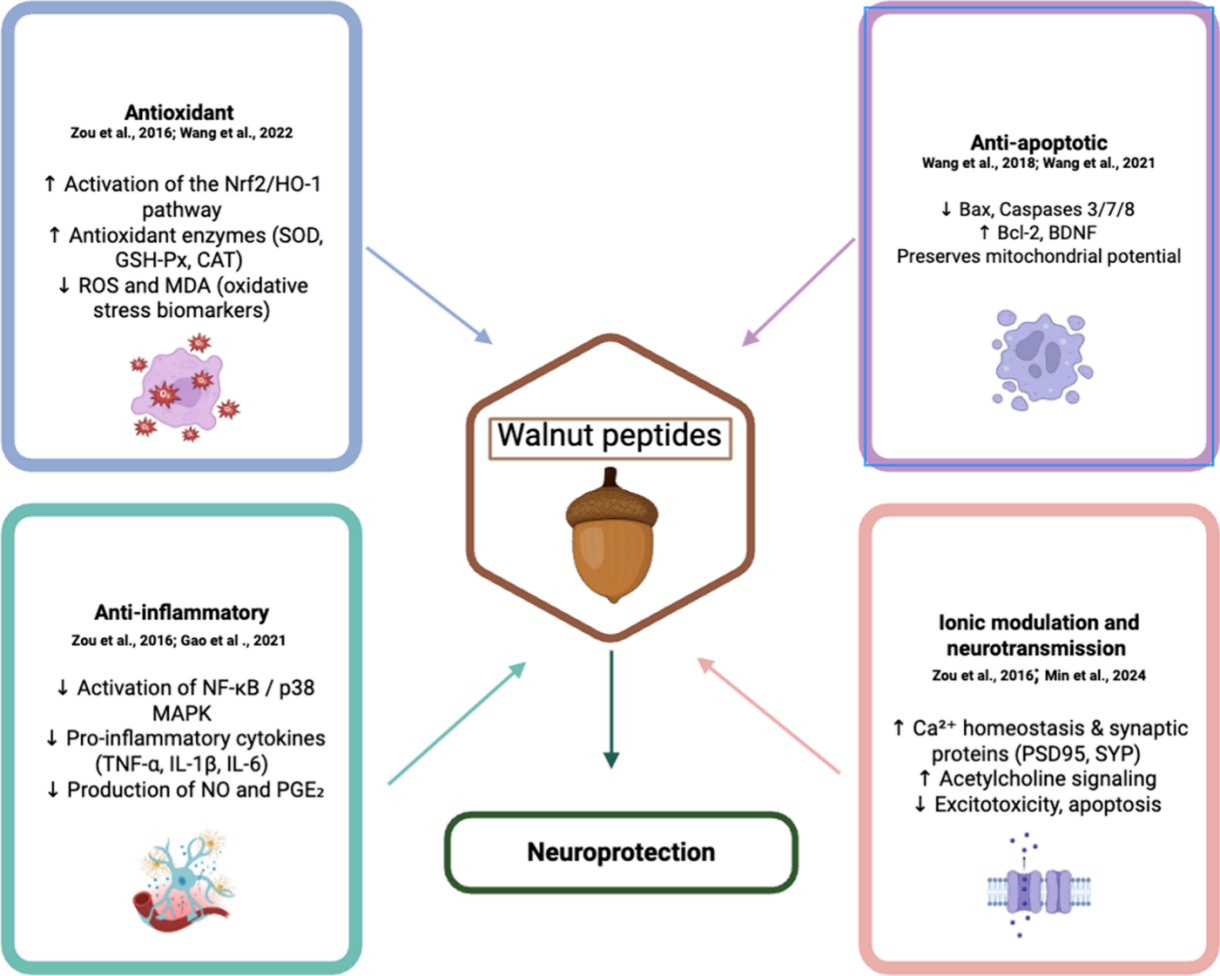
Neuroprotective mechanisms of walnut-derived peptides. The schematic diagram illustrates the main pathways through which walnut-derived peptides exert their neuroprotective effects.

In another study, cysteine-rich peptides with glycosylation modifications extracted from *A. bidentata* demonstrated neuroprotective capacity against NMDA-induced damage comparable to the positive control. The peptide was able to significantly increase the viability of HT22 cells exposed to NMDA, and the cells were able to proliferate even in the presence of NMDA when treated with the peptide.^38,39^

### 3.7 Use of Molecular Modeling and In Silico Techniques for Optimization of Interactions with Neuronal Targets

In silico approaches have proven to be powerful in predicting effects across various fields of study. Several articles have employed computational approaches to predict targets related to neuroprotection. A study used this approach to predict the targets of the peptide YYLLVR.^55^ Using tools such as PharmMapper and SwissTargetPrediction to identify targets and STRING and AutoDock Vina to analyze their interactions, the authors identified proteins such as ACE, ACE2, and AGTR1, suggesting a neuroprotective function against hypertension-induced damage. The group also experimentally confirmed these predictions using HUVEC and HT-22 cells treated with Ang II, observing decreased expression of ACE, ACE2, and AGTR1, which would normally be upregulated in these modified cell lines, further indicating potential neuroprotective activity.^55^

Molecular docking tests were conducted using Schrödinger tools to evaluate the binding potential of the peptides APCPNR, LGLFR, LIPQE, and SISWSS, isolated from *C. ternatea*, with AChE and beta-amyloid proteins, which are commonly associated with AD.^39^ All peptides tested showed binding potential with these targets, suggesting protective effects against the disease. Molecular docking was also performed using the HPEPDOCK tool on various peptides extracted from soybean.^26^ The aim was to evaluate their inhibitory activity against monoamine oxidase (MAO), which is also an important marker for neurodegenerative diseases such as AD. Peptides with the highest docking scores were synthesized and subjected to an MAO inhibition assay, where the in silico results were validated, showing that peptides YSPYPQ and PLYSN, which achieved the highest scores, indeed exhibited strong MAO inhibitory potential and, therefore, nutraceutical potential in the treatment of degenerative diseases.^26^

The neuroprotective potential of peptides found in corn gluten meal was evaluated. In this case, the approach was not exploratory as in the studies mentioned above. The peptides ALLTLSPLGPA and SPLGPA were subjected to in vitro tests that confirmed their inhibitory activity against prolyl oligopeptidase (POP).^61^ This enzyme degrades neuroactive molecules and is linked to neurocognitive disorders. After their activity was confirmed, molecular docking methodology was applied to better understand the mechanism of inhibition. Using AutoDock Vina software, the authors were able to demonstrate the peptide binding site on POP and confirm the noncompetitive nature of the inhibition. This approach demonstrates the versatility of computational tools in optimizing the search for neuroprotective peptides.^61^

Computational tools were also demonstrated for visualizing the mechanism of action of peptides. In this study, the peptide TWLPLPR (Tw-7) was evaluated for its neuroprotective effect against synaptic dysfunction caused by the protein C1q.^20^ After in vitro confirmation of its activity, the GROMACS tool was used to optimize the peptide’s molecular structure, docking was performed with AutoDock Vina, and the binding site was analyzed using PyMOL and LigPlus. This approach allowed the authors to elucidate the binding site and, therefore, the inhibitory mechanism of the peptide. The peptide Tw-7 was also evaluated to investigate its inhibitory activity on the protein MMP-9.^21^ PEP-FOLD3 was used to obtain the peptide structure, and AlphaFold2 for the target protein structure, with docking performed in Schrödinger Maestro. The results demonstrated that the peptide binds to the active site of MMP-9, inhibiting its function.

The PeptideRanker tool was used to predict the bioactivity of peptides containing arginine residues present in walnut proteins.^39^ Peptides with the highest predicted bioactivity were synthesized and evaluated for their neuroprotective effects, demonstrating positive results. Furthermore, in a subsequent study, simulated enzymatic hydrolysis was performed using the BIOPEP web platform to propose efficient methodologies for the production of these peptides.^10^ The enzymes trypsin and thermolysin showed high rates of release of arginine-containing peptides.

### 3.8 Safety Profile

Safety evaluation is an essential aspect for the therapeutic application of neuroprotective peptides. Among the reviewed studies, some provided relevant data regarding the toxicity profiles of these molecules (Figure 6). It was demonstrated that the peptide YYLLVR, at concentrations up to 200 μmol/L, showed no toxic effects on HT-22 cells, did not interfere with cell proliferation, and did not induce morphological alterations.^55^ Similar results were observed for the peptide BSP1, which at concentrations up to 300 μM did not affect HT-22 cell viability, indicating low toxicity within this range.^29^

**Figure 6:**
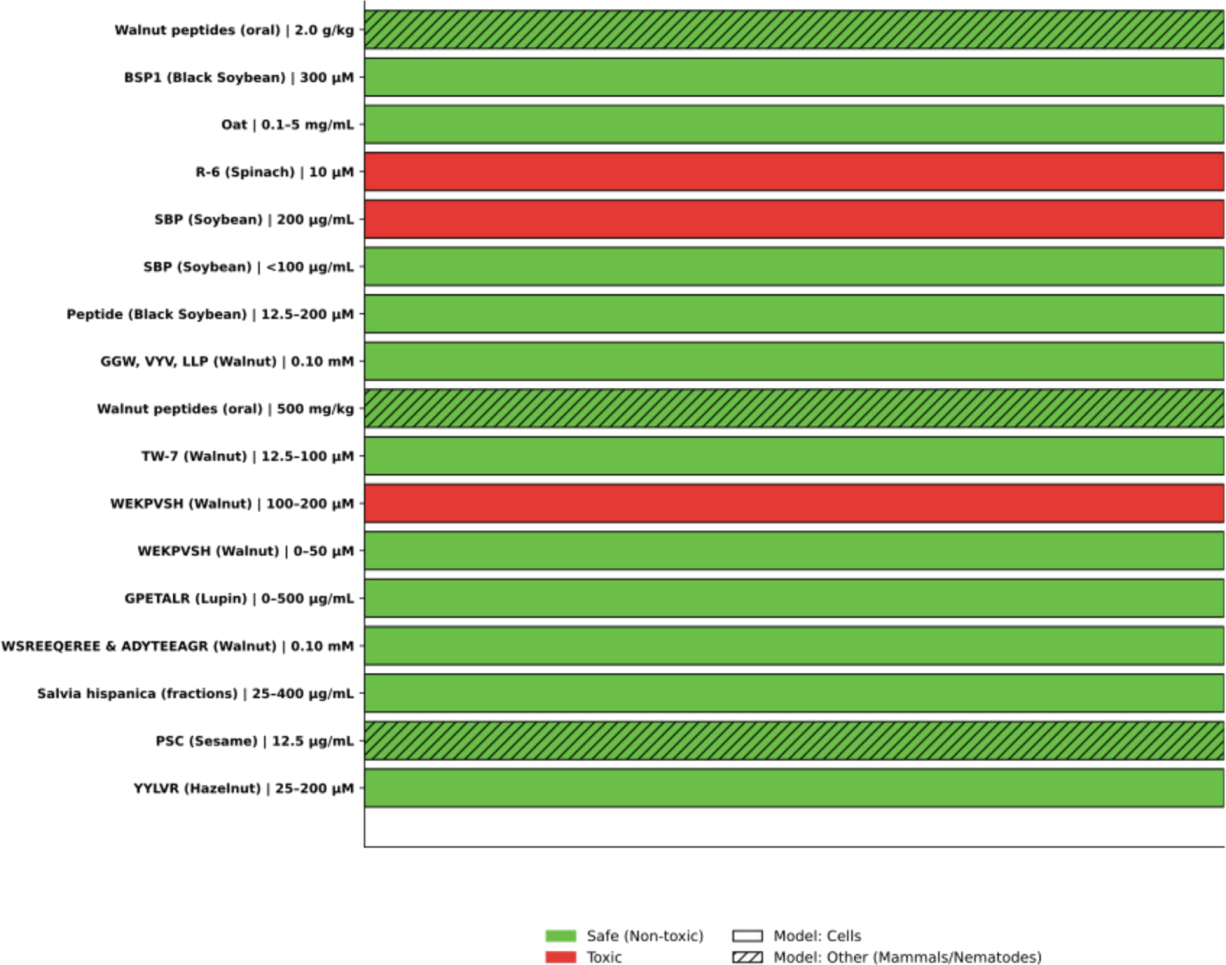
Toxicity assessment of plant-derived peptides with neuroprotective potential.

Peptides derived from walnuts, such as GGW, VYY, and LLPF, demonstrated a favorable safety profile in PC12 cells, even showing a significant increase in cell viability at concentrations of 0.10 mM. Another peptide, TW-7, showed no toxicity in bEnd.3 endothelial cells when tested at concentrations ranging from 12.5 to 100 μM after 24 h of treatment.^21,10^ The peptide WEKPPVSH, also extracted from walnuts, did not affect the viability of BV-2 microglial cells at concentrations of 0, 12.5, 25, and 50 μM.^41^ Similarly, peptides extracted from chia demonstrated cell viability above 80% in HMC3 microglial cells, indicating low cytotoxicity.^34^

In vivo models also support the safety of these peptides. For instance, acute toxicity studies in mice indicated that doses of walnut-derived peptides up to 5000 mg/kg did not cause mortality or adverse effects, with an LD50 above this dose.^48^ Similarly, administration of 20.1 g/kg of peptides derived from black soybean in mice did not result in mortality or significant adverse effects.^67^ Additionally, in *C. elegans*, peptides obtained from sesame cake increased the longevity and motility of the nematodes without causing negative effects, reinforcing their safety profile and therapeutic potential.^30^ Collectively, these findings underscore the therapeutic potential of neuroprotective peptides, evidencing their biocompatibility and low risk of toxicity at the tested concentrations.

However, despite the promising in vivo and in vitro results, translating these data to humans requires caution, as cellular and animal models do not fully replicate human metabolic and immune complexity. Therefore, clinical studies are essential to confirm the safety and efficacy of these peptides in prolonged treatments (Figure 6).

## 4 Conclusions and Perspectives

This Perspective provides a comprehensive synthesis of the current literature on plant-derived peptides with potential neuroprotective effects. The analyzed studies demonstrate that these compounds act through multiple mechanisms, including attenuation of oxidative stress, modulation of neuroinflammation, regulation of apoptosis, maintenance of ionic homeostasis, inhibition of toxic protein aggregation, and promotion of synaptic plasticity. In silico approaches have also significantly contributed to the prediction and understanding of interactions between peptides and their molecular targets. The diversity of plant sources, structural variety of the peptides, and experimental models, along with most toxicological tests indicating favorable safety profiles with low toxicity at the evaluated concentrations, reinforce the therapeutic potential of these compounds against neurodegenerative diseases.

However, there is a predominance of in vitro studies, highlighting the need for more in-depth investigations using complex biological models. Furthermore, aspects such as stability, bioavailability, BBB permeability, and pharmacokinetic profiles remain poorly explored, limiting progress toward clinical application. Methodological heterogeneity among studies and the scarcity of long-term functional assessments also compromise the comparability and robustness of the evidence. Therefore, future efforts should focus on standardizing methods, elucidating molecular mechanisms, and conducting well-designed toxicological and clinical studies. Such advances are crucial to establish plant-derived peptides as promising candidates for the treatment of neurodegenerative diseases.

## Supplementary Material


