# Supplementary material for: Plant-Derived Peptides with Neuroprotective Activity: Advances and Perspectives in the Prevention of Neurodegenerative Diseases
Source: ACS Omega. 2026 Apr 8;11(15):22458–78. doi: 10.1021/acsomega.6c00495 (PMC13103847; doi:10.1021/acsomega.6c00495)
Supplement: Supplementary file 1 [file ao6c00495_si_001.pdf]

Supplementary material

Plant-derived peptides with neuroprotective activity: advances and perspectives in the prevention of neurodegenerative diseases

Maria Eduarda Maia, Matheus Carvalho, Cleyton Sousa Gomes, Milena Arruda, Ana Júlia Antunes de Magalhães, Davi Farias

Supplementary material 1A: List of abbreviations

**6-OHDA**	6-hydroxydopamine
**Aβ**	Beta-amyloid peptide
**ABTS.**	2,2′-azino-bis(3-ethylbenzothiazoline-6-sulfonic acid)
**ACh**	Acetylcholine
**AChE**	Acetylcholinesterase
**AD**	Alzheimer's disease
**Ang II**	Angiotensin II
**APP/PS1**	Amyloid precursor protein/presenilin 1 transgenic model
**Bax**	Bcl-2-associated X protein (pro-apoptotic protein)
**BBB**	Blood–brain barrier
**BDNF**	Brain-derived neurotrophic factor
**CAT**	Catalase
**DPPH**	2,2-diphenyl-1-picrylhydrazyl
**GPX**	Glutathione peroxidase
**GSH**	Reduced glutathione
**GST**	Glutathione S-transferase
**H2O2**	Hydrogen peroxide
**HD**	Huntington's disease
**HUVEC**	Human umbilical vein endothelial cells
**LDH**	Lactate dehydrogenase

**LPS**	Lipopolysaccharide
**MAO-A**	Monoamine oxidase A
**MAO-B**	Monoamine oxidase B
**MDA**	Malondialdehyde
**MMP**	Mitochondrial membrane potential
**NF-κB**	Nuclear factor kappa B
**NO**	Nitric oxide
**Nrf2**	Nuclear factor erythroid 2–related factor 2
**ORAC**	Oxygen radical absorbance capacity
**PC12**	Rat pheochromocytoma cell line
**PD**	Parkinson's disease
**ROS**	Reactive oxygen species
**SH-SY5Y**	Human neuroblastoma cell line
**SKN-1**	SKN-1 transcription factor (homolog of mammalian Nrf2)
**SOD**	Superoxide dismutase
**TNF-α**	Tumor necrosis factor alpha
**WPHL**	Walnut protein hydrolysate
